# Supplementary material for: Droplet-based screening of phosphate transfer catalysis reveals how epistasis shapes MAP kinase interactions with substrates
Source: Nat Commun. 2022 Feb 11;13:844. doi: 10.1038/s41467-022-28396-4 (PMC8837617; doi:10.1038/s41467-022-28396-4)
Supplement: Supplementary file 1 — Supplementary Information [file 41467_2022_28396_MOESM1_ESM.pdf]

Supplementary Materials for

Droplet-based screening of phosphate transfer catalysis reveals how epistasis shapes MAP kinase interactions with substrates

Authors: Remkes A. Scheele[1†], Laurens H. Lindenburg[1†], Maya Petek[1,2†], Markus Schober[1], Kevin N. Dalby[3] and Florian Hollfelder[1*]

* Correspondence to: fh111@cam.ac.uk, tel: +44 (0)1223 766048.

This PDF file includes:

Supplementary Notes 1-3
Supplementary Figures 1 to 25
Supplementary Tables 1 to 8
Supplementary References

Supplementary Note 1. Filtering the variants detected in the high gate to identify the set of active variants

The different MKK variants in the SpliMLiB library vary in activity, whether through a change in binding affinity of the target peptide substrate or by a change in phosphorylation kinetics in the MKK active site. The sum of this activity is measured as a single point readout, the amount of retained GFP on the beads, which translates into the green fluorescent signal during FACS bead sorting.

It has been noted in the main text that the beads carrying the negative control variant ^{ca}*MKK1*$^{I9A/L11A}$ show *impaired* activation, such that the majority of beads show a low GFP signal in flow cytometry (**Figure 2**). However, a small proportion of individual beads still appear in the medium and high gates. Conversely, the WT beads largely appear in the high gate, but also the medium and low gates. The split of WT MKK1 between the high and medium gates is by design, since the medium/high gate boundary was set to the *median* signal of WT ^{ca}*MKK1* beads – thus half of the beads in WT are expected to fall in the lower two gates.

As a result of these considerations, each variant in the SpliMLiB library shows a distribution in the sorting experiment, which translates in a distribution of sequencing read counts. Mere detection of a variant in a single gate is not sufficient to assign it particular activity; for example, WT ^{ca}*MKK1* has 51 reads in the high gate, 36 in medium gate and 34 in low gate. The negative control variant ^{ca}*MKK1*$^{I9A/L11A}$ primarily appears in the low gate with 20 reads, but also has 6 reads in the medium gate and 0 in the high gate.

The majority of conclusions in the main analysis rely on a low rate of false positives in the set of active variants, which requires a conservative (high read count) cutoff for identifying positive variants. Inspection of the data in **Supplementary Figure 13** suggest a value between 30 and 100 may be appropriate.

We choose to build the set of active variants by focusing on the variants that show a sequencing count profile that is similar to WT or even more enriched in the high gate. Specifically, that requires:

- 51 or more reads in the high gate, as WT MKK1 has 51 reads; this identifies 29,603 variants.
- More reads in the high gate than in the medium or in the low gate (H>M and H>L count); this removes 35 variants are more abundant in the lower gates.
- At least 42% of reads in the high gate out of total, removing further 4 variants.
- No stop codons, which may occur as early PCR errors during sequencing; removes one last variant.

Together, this filtering strategy generates a set of 29,563 variants that we confidently describe as a set of active variants.

Supplementary Note 2. Reflection on options for variant counting (defining active variants) and discussion of significance.

In this manuscript, we present an analysis of the active MKK1 variant sequences *as a group*; each sequence is weighed equally, regardless of its abundance (i.e. sequencing count) in the high activity FACS gate. Consequently, the outcome of the analysis must depend on which sequences are included in this analysis. This raises the questions which options are available for defining activity from FACS data?
There is some correlation between count and activity (**Supplementary Figure 10**), but we have to few gates to resolve the activity spectrum, especially for variants of medium activity in a meaningful way.

The first set of thresholds we set is used in **Figure 3a** for the purpose of counting the number of variants we detected at all. Here we explain our choices:

- High gate: for the purpose of counting variants that are observed (but without inferring activity, yet) we use a cut-off of 10 or more reads in the high gate, which removes the sequencing noise with some margin. Here, the cutoff can be relatively high, since this gate contains a minority of library variant and is sequenced more deeply than the other two.
- Medium gate: there is a larger proportion of less well sequenced variants, so lowering the detection limit below 10 is appropriate to increase coverage. The number of detected variants stabilises at 5 reads / variant.
- Low gate: a similar trend to the medium gate is observed here, with the distribution of variants per read shift even more towards low sequencing counts per variant. In order to maximise the discovery of variants, we choose to use the cut-off of 3 reads per variant.

However, just because a variant appears in a given gate, its occurrence does not confirm that this is the actual activity. Indeed, the high gate has a known 8% false positive rate and the low gate a 50% false negative rate, so clearly some further consideration is necessary.

We explored two ways of defining active variants.

1. **A minimum required number of sequencing reads in the high gate**

This consideration is based upon the observation that variants that are truly enzymatically active will be abundant in the high gate, while false positives appear in the high gate only rarely. The two controls have the following distribution:

WT: H 51, M 36, L 34

Neg: H 0, M 6, L2

This shows that active variants may appear in the lower gates, but inactive variants should be rare in the high gate. However, it is difficult to set a clear threshold based on only two datapoints with very different distributions.

Option 1: setting the high gate cut-off at 10 (as in the Venn diagram) as the only criterion for activity, while accepting a possibly higher proportion of false positives in this set. This set is the largest and the most uncertain, giving 36K variants.

Option 2: setting the high gate cut-off at 10 (as in the Venn diagram) and then performing additional filtering, throwing out the variants that are common in the lower gates. This filtering is necessary, since a variant might just happen to be abundant in multiple gates (uncommon due to even composition of the starting library, but still possible). Such filtering reduces the variant count by 4k: the cutoff alone identified 36K variants in the high gate, which is reduced to 32K for the main analysis.

This scheme shows reasonable classification of point mutants, where the WT sequence is identified on the lower end of the distribution compared to ‘active’ single mutants. However, this scheme does have the drawback that the filtering step is once again arbitrary and possibly quite complicated.

Option 3: setting the cut-off higher, at 50 or even 100. Since most variants in the lower gates appear at lower sequencing counts, this would achieve most of the cleanup from the previous approach. It is also methodologically sounder, since fewer parameters need to be chosen manually (one number compared to several choices in option 2).

We evaluated how the choice of the cutoff affects the number of selected variants (**Supplementary Figure 14**), which demonstrates that there isn't an obvious choice (flat line). Instead, we can only justify the choice by confirming that the chosen value does not affect conclusions.

Further examination of the dataset showed that most of the complicated filtering (option 2) affects variants below a threshold of 50 reads in the high gate (WT has 51 reads). By using a cutoff that is referenced on the WT counts, we both have a data-supported cutoff point and avoid the complicated filtering: by using a higher cutoff of 50 we can simplify that part and only lose about 3K variants.

2. **A minimum required proportion of sequencing reads in the high gate**

Alternatively, we could also define variant activity according to the distribution of sequencing reads across the three FACS/NGS bins, rather than considering high gate sequencing only. However, because the high gate was set in a conservative fashion, a filtering scheme is once again necessary. While the dataset certainly contains variants with >80% or >90% reads in the high gate, there are certainly other variants that are also active (WT only has 42% total reads in the high bin).

While testing the robustness of our conclusions, we did construct such a dataset, again using the WT read distribution as a reference. In that analysis we included variants that had >40% reads in the high gate and <30% in the low gate, which again yielded 36K variants.

We examined the single mutant placement, amino acid enrichment and epistasis patterns in all of these constructions and found that only minimal numeric differences appear (**Supplementary Figure 15**). The enrichment pattern, epistasis trends and the Φ-X-Φ motif are reliably identified in all versions of the analysis. Here we present the figures and the sequence similarity network based on the most conservative choice (number of reads in the high gate equal or higher to the WT 51 reads), which is representative of alternative definitions of the active dataset.

Supplementary Note 3. A guide to quantification of epistatic interactions in D-domains

In this manuscript, (intra-gene) epistasis formally refers to deviation from linearity when examining the effect of mutations at more than one position in the gene. The non-linearity can be understood in terms of Bayes' Law about conditional probability. Although the definitions stem from probability theory, here they are discussed in terms of *frequency* of the variant in questions – for our purpose, the frequency of each variant in the dataset and the expected probability are interchangeable.

In order to describe the quantitative analysis of intra-gene epistasis in the MKK1 D-domain, we need to differentiate between observed and expected variant frequencies.

Single position frequencies are calculated for each position independently, across the entire dataset.

- f_a is the frequency of the residue a at the position in question, where a may be any of the allowed residues (2, 12 or 13 different options, depending on the randomised position.
- f_a^{id} is the *ideal* frequency of the residue a at a given position, that is one predicted by an uniform distribution. Therefore, f_a^{id} is the same for all a at a

given library position, and equals $\frac{1}{2}$ at position 8a, $\frac{1}{13}$ at position 7a and $\frac{1}{12}$ at position 6, 9, 11 and 13. $\sum_a f_a^{id} = 1$.

- f_a^{obs} is the *observed* frequency the residue a at the position in question. It is calculated from the final active dataset of active variants by counting the number of times a particular residue appears in the active variants. It differs from f_a^{id} because of true enrichment of active variants in the dataset, as well as sampling effects and experimental noise. $\sum_a f_a^{obs} = 1$.

The change in single position frequencies, $\frac{f_a^{obs}}{f_a^{id}}$, is displayed in heatmap format in **Figure 3b.** A logarithmic scale is used to equally display fold changes in both direction, both for enrichment and depletion of particular residues.

When considering two positions at once ($f_{a,b}$, where a and b denote the residue identities in the two positions), several frequencies are of interest. Before giving the specific definitions, note that Bayes' Law in this notation states that $f_{a,b} = f_{a|b} \cdot f_b$. If the $f_{a|b} = f_a$ (the condition for independence), that is the frequency of residue a at one position does not change depending on the residue b at the other position, can we calculate the joint frequency directly. If this equality does not hold, the system is non-linear and epistasis is present.

Two position definitions:

- Analogous to the single position definition, $f_{a,b}^{id}$ is the *ideal* frequency of the residues a, b at the given positions. Since the single position ideal frequencies are known and the positions are randomised independently, $f_{a,b}^{id} = f_{a|b}^{id} \cdot f_b^{id} = f_a^{id} \cdot f_b^{id}$. Similarly, these frequencies (like any well-defined probabilities) sum to 1: $\sum_{a,b} f_{a,b}^{id} = 1$, but only when the sum is done across both randomised positions.
- $f_{a,b}^{obs}$ is the *observed* frequency of the residues a, b at the two positions in question. It is calculated from the final active dataset of active variants by counting the number of times a particular residue appears in the active variants. It differs from f_a^{id} because of true enrichment of active variants in the dataset, as well as sampling effects and experimental noise. $\sum_a f_a^{obs} = 1$.

The change in two position frequencies, $\frac{f_{a,b}^{obs}}{f_{a,b}^{id}}$, is displayed in heatmap format in **Figure 4.** A logarithmic scale is used to equally display fold changes in both directions, both for enrichment and depletion of particular residues. Thus, the Figure 4 heatmap is analogous to Figure 3B, in that is shows the active variant deviation from an ideally balanced library. While useful for orientation, in itself it does not prove the presence of epistasis.

A map of epistasis is derived from the observed two-position (joint) frequency, compared to the expected joint frequency – this time, the expectation comes from single position frequencies. Specifically, if there is no epistasis, the joint frequency should be the product of the two single position frequencies: $f_{a,b}^{obs-indep.} = f_a^{obs} \cdot f_b^{obs}$. However, if epistasis is present, then this equality does not hold. Therefore, the map of epistasis in **Supplementary Figure 17** shows

$$\frac{f_{a,b}^{obs}}{f_{a,b}^{obs-indep.}} = \frac{f_{a,b}^{obs}}{f_a^{obs} \cdot f_b^{obs}}.$$

Sometimes, it is easier to identify how the presence of a particular residue b at a distant position influences the single position preferences (f_a) at the first position. In that situation, we need to examine $\frac{f_{a|b}^{obs}}{f_a^{id}}$; as a single position preference, normalising to the uniform distribution preference is appropriate. When placed on a logarithmic scale, this generates the heatmaps in **Supplementary Figure 18** – each square examines a pair of positions so that each choice of b is a column in the appropriate square.

A

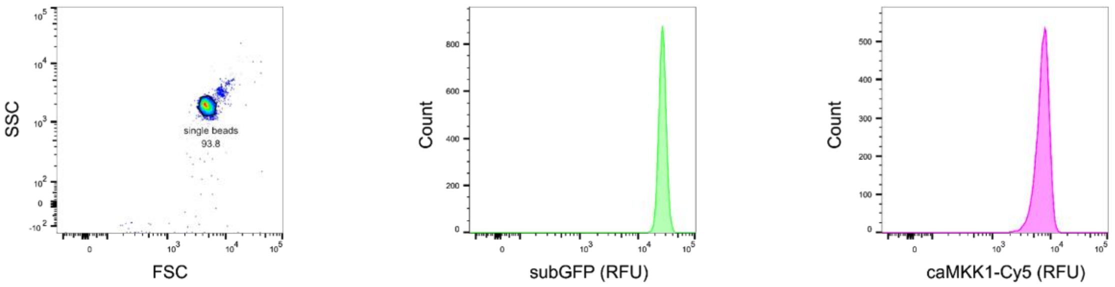

SampleID	#Beads analysed	Single Beads (%)*	Median: subGFP	Mean: subGFP	Median: subGFP	Mean: subGFP
^{ca}MKK1	10,101	94	26,969	27,196	7,472	7,397

* of total number of beads analysed.

B

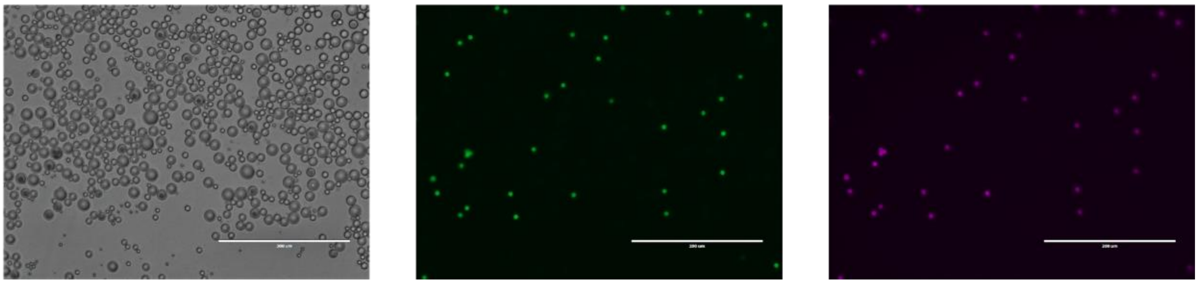

Supplementary Figure 1. Functionalisation of paramagnetic beads and emulsification.

(A) Flow cytometric analysis of single beads shows homogeneous coating with subGFP (middle) and ^{ca}*MKK1*-Cy5 (right). (B) Emulsification of the functionalised beads visualised by microscopy: a bright-field image of the emulsified beads (left), and fluorescent channels for subGFP (middle) and ^{ca}*MKK1*-Cy5 (right). The micrographs show a typical emulsion (see other emulsions in **Supplementary Figure 2**). The emulsification process has been repeated >30 times with similar results.

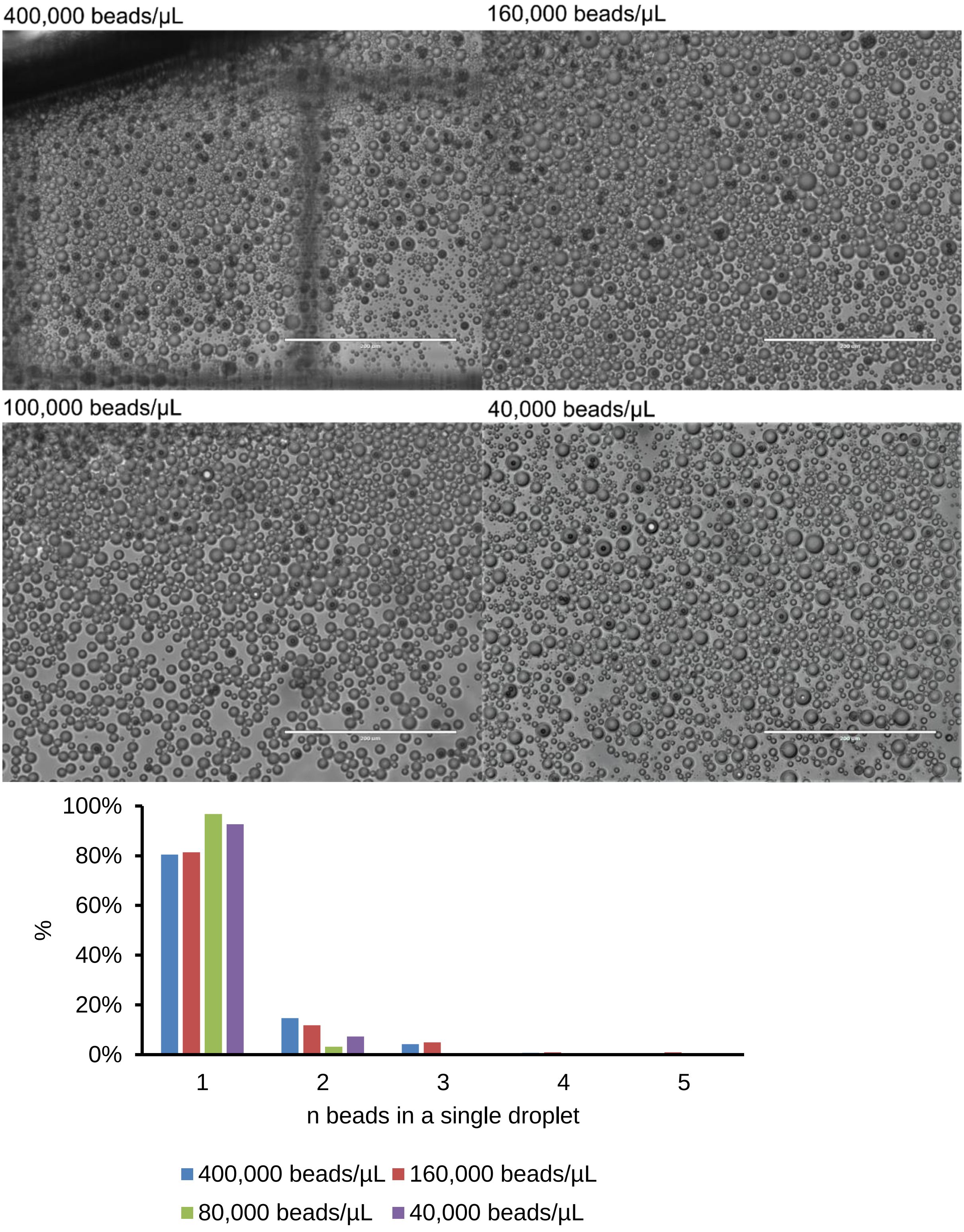

Supplementary Figure 2. Optimisation of the number of beads per droplet. Following a previously established emulsification protocol[1] (12.5 µL IVTT/ERK2 mixture + 100 µL 1% (v/v) RAN in HFE-7500), different numbers of beads were encapsulated. As double or higher-order encapsulations decrease the reliability of the screen by increasing the false positive rate, all experiments were carried out using 40.000-80.000 beads/µL (500,000-1,000,000 beads/emulsion).

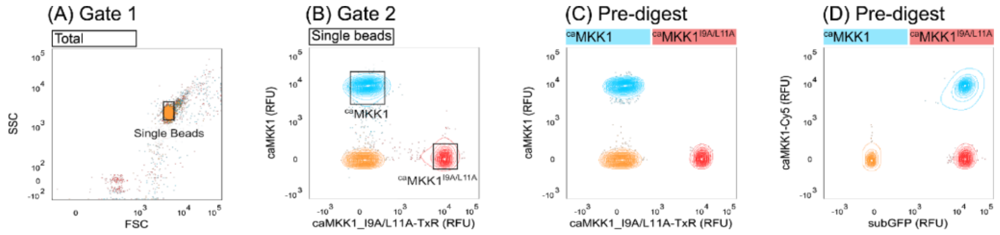

Figure	SampleID	#Total Beads analysed	Single Beads (%)*	MKK1 mutant	MKK1 mutant (%)**	Median: subGFP	Mean: subGFP	Median: Cy5	Mean: Cy5	Median: Texas-Red	Mean: Texas-Red
A-D	Non-Funct.	5,046	94	Non-Funct.	100	-2	-2	-23	-17	135	132
A-D	caMKK1	5,186	91	caMKK1	97	19,121	19,369	7,801	7,749	191	194
A-D	caMKK1$^{I9A/L11A}$	5,323	89	caMKK1$^{I9A/L11A}$	97	19,352	19,596	0	8	13,752	13,796

* of total number of beads analysed. ** of single beads

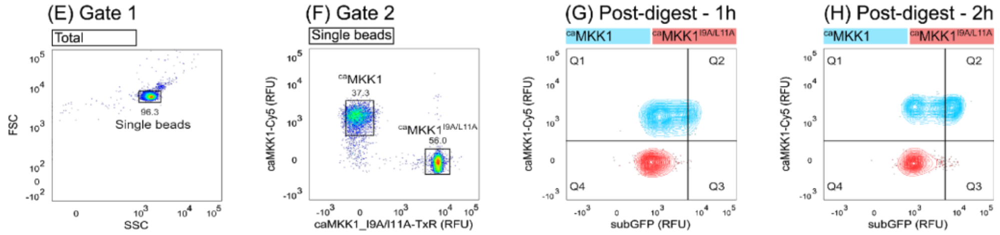

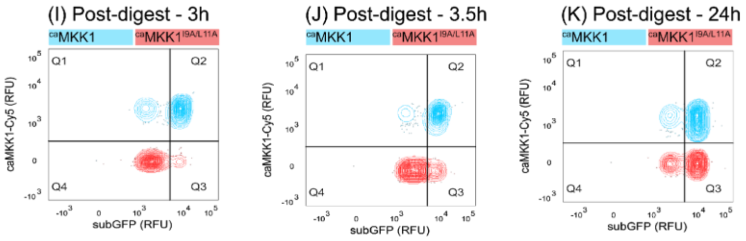

Figure	SampleID	#Total Beads analysed	Single Beads (%)*	MKK1 mutant	MKK1 mutant (%)**	Q1 (%)***	Q2 (%)***	Q3 (%)***	Q4 (%)***
E-G	1h	10,092	96	caMKK1	37	91	9.2	0	0
				caMKK1$^{I9A/L11A}$	56	0	0	0.1	100
H	2h	10,144	95	caMKK1	39	50	50	0	0
				caMKK1$^{I9A/L11A}$	57	0	0	0.5	100
I	3h	10,195	95	caMKK1	36	9.0	91	0	0
				caMKK1$^{I9A/L11A}$	59	0	0	4.0	96
J	3.5h	10,098	95	caMKK1	36	4.0	96	0	0
				caMKK1$^{I9A/L11A}$	59	0	0	13	87
K	24h	10,162	92	caMKK1	35	7.2	93	0	0
				caMKK1$^{I9A/L11A}$	39	0	0	83	17

* of total number of beads analysed. ** of single beads. *** of MKK1 mutant (caMKK1 or caMKK1$^{I9A/L11A}$) beads.

Supplementary Figure 3. Time dependence of ERK2 activation by ^{ca}MKK1 or binding impaired ^{ca}MKK1$^{I9A/L11A}$.

(A-C) Gating strategy for functionalisation of two separate bead populations with either ^{ca}*MKK1*-Cy5 (aqua) or ^{ca}*MKK1*$^{I9A/L11A}$-TxR (red) DNA compared to non-functionalised beads (orange). (D) Functionalisation of both bead populations with equal amounts of subGFP compared to non-functionalised beads (orange). (E-F) Gating strategy for mixtures of ^{ca}*MKK1* and ^{ca}*MKK1*$^{I9A/L11A}$ beads. (G-K) Beads carrying ^{ca}*MKK1* or ^{ca}*MKK1*$^{I9A/L11A}$ were mixed 1:1 and emulsified. The emulsion was incubated for the time shown above the plots. After de-emulsification and digestion with chymotrypsin, the bead mixture was analysed by flow cytometry, differentiating the two genotypes through TxR/Cy5 fluorescence. Analysis of the bi-modal distribution of beads with phosphorylated subGFP (Q2 or Q3) or non-phosphorylated subGFP (Q1 and Q4) shifting over time indicated ^{ca}MKK1 to be more efficient at phosphorylation of ERK2 than ^{ca}MKK1$^{I9A/L11A}$. The kinetic resolution for D-domain complementarity is best resolved when incubating the beads for 3 hours: Q2 (true positives) =91%, Q3 (false positives) =4.0%.

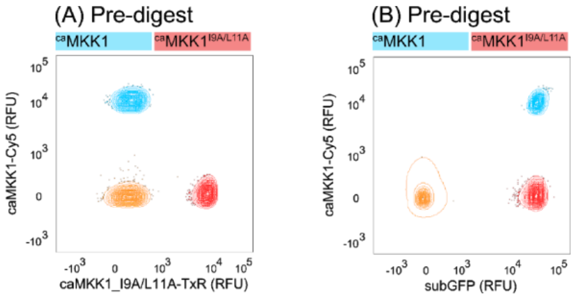

Figure	SampleID	#Total Beads analysed	Single Beads (%)*	MKK1 mutant	MKK1 mutant (%)**	Median: subGFP	Mean: subGFP	Median: Cy5	Mean: Cy5	Median: Texas-Red	Mean: Texas-Red
A and B	Non-Funct.	10,302	57	Non-Funct.	100	57	24	-20	-11	296	296
A and B	^{ca}MKK1	11,291	75	^{ca}MKK1	87	48,502	49,038	10,045	10,242	346	349
A and B	^{ca}MKK1$^{I9A/L11A}$	12,465	66	^{ca}MKK1$^{I9A/L11A}$	80	45,419	44,857	32	29	10,381	10,235

* of total number of beads analysed. ** of single beads

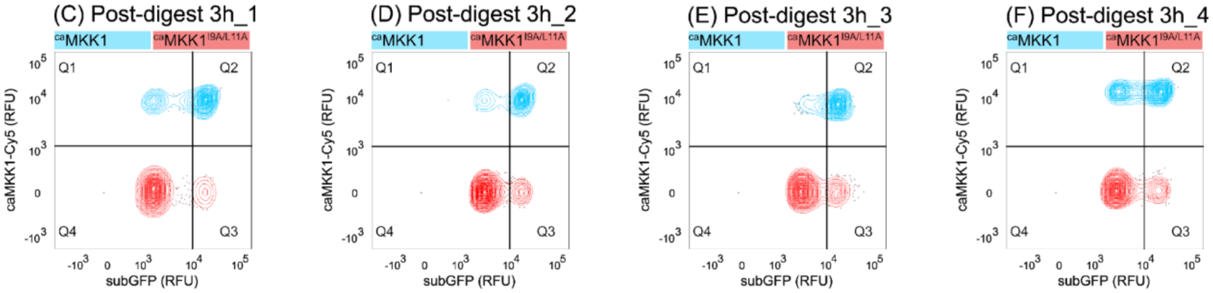

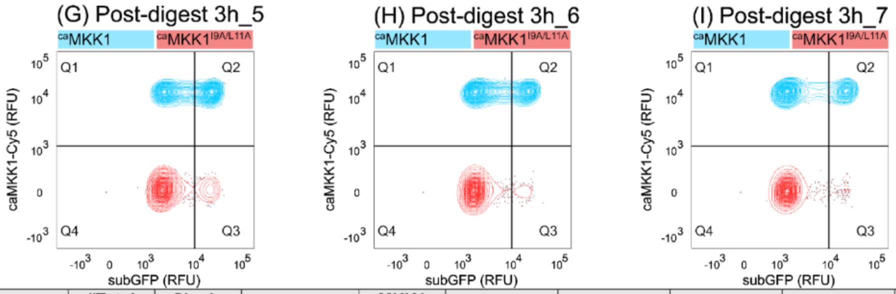

Figure	SampleID	#Total Beads analysed	Single Beads (%)*	MKK1 mutant	MKK1 mutant (%)**	Q1 (%)***	Q2 (%)***	Q3 (%)***	Q4 (%)***	PPV (%)****
C	3h_1	11,062	83	^{ca}MKK1	47	13	87	0	0	94
				^{ca}MKK1$^{I9A/L11A}$	48	0	0	5.3	95	
D	3h_2	11,456	78	^{ca}MKK1	45	6.1	94	0	0	93
				^{ca}MKK1$^{I9A/L11A}$	50	0	0	7.5	93	
E	3h_3	10,154	95	^{ca}MKK1	44	8.0	92	0	0	90
				^{ca}MKK1$^{I9A/L11A}$	35	0	0	10	90	
F	3h_4	10,092	92	^{ca}MKK1	39	27	73	0	0	89
				^{ca}MKK1$^{I9A/L11A}$	55	0	0	9.3	91	
G	3h_5	10,060	94	^{ca}MKK1	41	44	56	0	0	92
				^{ca}MKK1$^{I9A/L11A}$	54	0	0	5.2	95	
H	3h_6	10,173	90	^{ca}MKK1	42	70	30	0	0	93
				^{ca}MKK1$^{I9A/L11A}$	54	0	0	2.3	98	
I	3h_7	10,061	94	^{ca}MKK1	43	78	22	0	0	92
				^{ca}MKK1$^{I9A/L11A}$	54	0	0	1.8	98	
AVERAGE				**^{ca}MKK1**		**35 ± 27**	**64 ± 27**	-	-	**92 ± 2**
				**^{ca}MKK1$^{I9A/L11A}$**		-	-	**6.0 ± 3.0**	**94 ± 2.9**	

* of total number of beads analysed. ** of single beads. *** of MKK1 mutant (^{ca}MKK1 or ^{ca}MKK1$^{I9A/L11A}$) beads. **** Positive Predictive value

Supplementary Figure 4. Reproducibility to enrich for ERK2-complementary D-domains.

(A) Functionalisation of two separate bead populations with either *^{ca}MKK1*-Cy5 (aqua) or *^{ca}MKK1$^{I9A/L11A}$*-TxR (red) DNA compared to non-functionalised beads (orange) (B) Functionalisation of both bead populations with equal amounts of subGFP compared to non-functionalised beads (orange). (C-I) Beads carrying *^{ca}MKK1* or *^{ca}MKK1$^{I9A/L11A}$* were mixed in a ration of 1:1 and emulsified. All individual emulsion populations were incubated for three hours. After de-emulsification and digestion with chymotrypsin, the bead mixture was analysed by flow cytometry, differentiating the two genotypes

through TxR/Cy5 fluorescence. Analysis of the bimodal distribution of beads with phosphorylated subGFP (Q2 or Q3) or non-phosphorylated subGFP (Q1 and Q4) for each sample (**Figure 2E)** showed the screen to robustly enrich for ^{ca}MKK1 over ^{ca}MKK1$^{I9A/L11A}$;

Positive predictive value is defined as: Q2/(Q2+Q3)
Average positive predictive value = 92±2%.

Coverage as a function of oversampling (X): 1-(Q1ˆX)
Average Q1 = 35%
Oversampling three-fold gives 0.96, or 96% coverage.

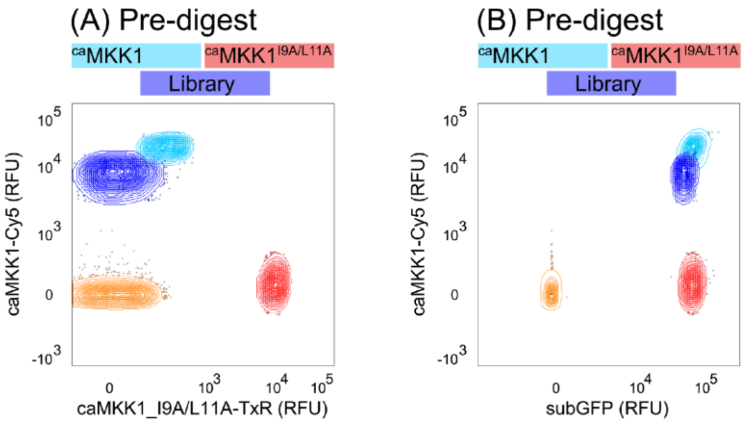

Figure	SampleID	#Total Beads analysed	Single Beads (%)*	MKK1 mutant	MKK1 mutant (%)**	Median: subGFP	Mean: subGFP	Median: Cy5	Mean: Cy5	Median: Texas-Red	Mean: Texas-Red
A and B	Non-Funct.	10,602	87	Non-Funct.	100	-8	-6	-14	-6	41	45
A and B	^{ca}MKK1	10,092	96	^{ca}MKK1	90	88,543	89,374	18,566	18,503	368	378
A and B	^{ca}MKK1$^{I9A/L11A}$	10,081	96	^{ca}MKK1$^{I9A/L11A}$	67	84,404	85,210	93	99	12,963	12,988
A and B	^{ca}MKK1 Lib.	10,257	89	^{ca}MKK1 Lib.	95	62,981	64,038	7,078	7,065	46	55

* of total number of beads analysed. ** of single beads

Supplementary Figure 5. Flow cytometric analysis of the SpliMLiB library and ^{ca}MKK1 / ^{ca}MKK$^{1I9A/L11A}$ control beads.

(A) The SpliMLiB synthesis[2] (dark blue) (strategy and oligonucleotides in **Supplementary Figure 25** and **Supplementary Table 6**) yielded a strong Cy5 signal compared to non-functionalised beads (orange) when ligating on the final fragment (fragP6X-cy5) - indicative of successful library synthesis. The same-day control samples of beads functionalised with full-length *^{ca}MKK1/^{ca}MKK$^{1I9A/L11A}$* amplicons are shown in aqua/red, respectively. (B) The average subGFP concentration on the library beads was slightly lower than the control beads (1.4x), which was taken into account when gating the library samples after chymotrypsin digest (**Supplementary Figure 7**).

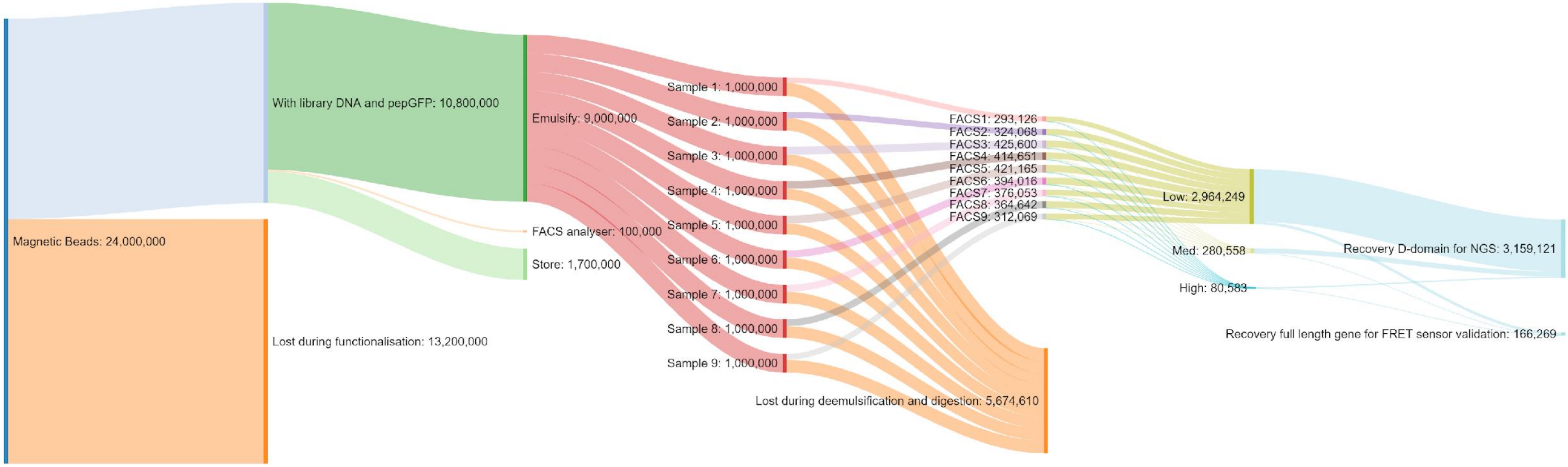

Supplementary Figure 6. Flow diagram of library bead preparation and screening.

A starting amount of 2.4 x 10^7 magnetic beads were functionalised stepwise through the SpliMLiB approach[2] and a final immobilisation of subGFP. During the process, 1.3 x 10^7 beads were lost in the washing steps, leaving 1.1 x 10^7 beads left to be screened. To accommodate screening of a maximum of 80.000 beads/µL (see **Supplementary Figure 2**) in the optimised emulsion of 12.5 µL IVTT/ERK2 and 100 µL oil, the SpliMLiB library was divided into 9 samples of 1E7 beads/emulsion. After incubation of the emulsions for 3 hours, the samples were de-emulsified, and chymotrypsin was added. 3.3 x 10^6 beads were retained through these processes and sorted by flow cytometry in parallel into Low/Medium/High gates. 95% of the sorted beads were used for recovery of the D-domain fragment for next-generation sequencing, while 5% of the sorted beads were used for recovery of the full-length *MKK1* gene for secondary validation with the FRET sensor.

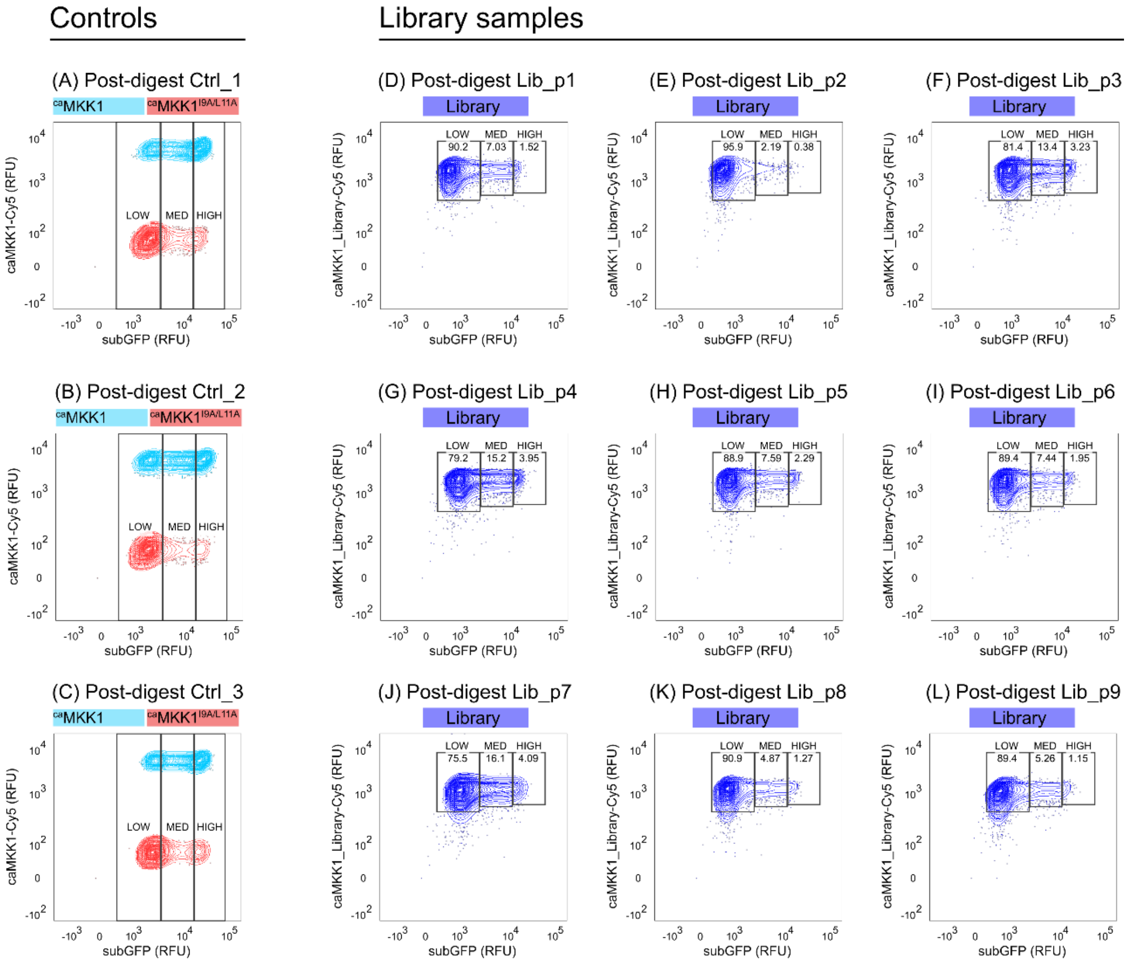

Figure	SampleID	#Total Beads analysed	Single Beads (%)*	MKK1 mutant	MKK1 mutant (%)**	HIGH (%)***	MED (%)***	LOW (%)***
A	Control 1	25,977	89	^{ca}MKK1	39	55	26	17
				^{ca}MKK1$^{I9A/L11A}$	55	4.5	9.6	85
B	Control 2	25,283	88	^{ca}MKK1	42	40	30	29
				^{ca}MKK1$^{I9A/L11A}$	55	2.8	5.7	91
C	Control 3	15,767	94	^{ca}MKK1	60	55	26	17
				^{ca}MKK1$^{I9A/L11A}$	32	7.2	14	78

* of total number of beads analysed. ** of single beads. *** of MKK1 mutant (^{ca}MKK1 or ^{ca}MKK1$^{I9A/L11A}$) beads.

Figure	SampleID	#Total Beads analysed	Single beads (%)*	HIGH (%)**	MED (%)**	LOW (%)**
D	Library part 1	233,303	90	1.5	7.0	90
E	Library part 2	325,959	90	0.4	2.2	96
F	Library part 3	429,757	88	3.2	13	81
G	Library part 4	415,884	89	4.0	15	79
H	Library part 5	421,158	90	2.3	7.6	89
I	Library part 6	394,016	85	2.0	7.4	89
J	Library part 7	10,863	91	4.1	16	76
K	Library part 8	237,911	92	1.3	4.9	91
L	Library part 9	356,420	89	1.2	5.3	89

* of total number of beads analysed. ** of single beads.

Supplementary Figure 7. Individual plots for all library samples and the three same-day controls.

All emulsions were incubated for three hours. (A-C) Three individual controls to set the gates on the library samples. (D-L) The nine library samples and their sorting gates after chymotrypsin digest. The absolute GFP-intensity gating windows were shifted 1.4-fold, taking into account the relative initial GFP intensities of *caMKK1-Cy5/caMKK1*$^{I9A/L11A}$ to the library carrying beads before chymotrypsin digestion (**Supplementary Figure 5**).

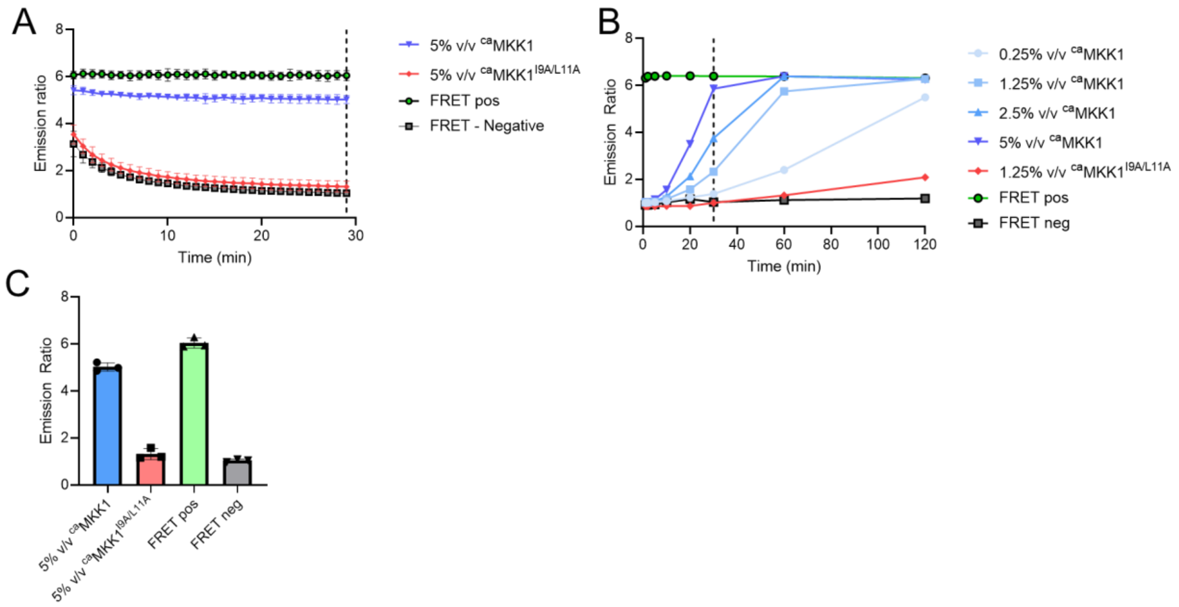

Supplementary Figure 8. Characterisation of the FRET sensor for secondary screening.

(A) The controls were incubated with chymotrypsin for 30 minutes, measuring the change in emission ratio.[3] Evidence for the FRET sensor's stability over time is shown as green circles. Addition of chymotrypsin to the FRET sensor cuts the ERK2 phosphorylation site between the two fluorescent proteins of the FRET sensor, resulting in maximal degradation of the FRET sensor after 30 minutes of incubation (black squares).[3] When the FRET sensor is incubated with 5% (v/v) IVTT expressed ^{ca}MKK1 (blue) or ^{ca}MKK1$^{I9A/L11A}$ (red) and ERK2 for half an hour prior to chymotrypsin digest (See Figure B), ^{ca}MKK1 will have phosphorylated most of ERK2 and consequently the FRET sensor, making it resistant to chymotrypsin digest. Standard deviations derived from three biological repeats are shown.

(B) Different volumes of IVTT expressed ^{ca}MKK1(blue) or ^{ca}MKK1$^{I9A/L11A}$ (red) was combined with purified ERK2, and the FRET sensor. After each time point, an aliquot was taken, and incubated with chymotrypsin for 30 minutes, with the resulting emission ratio plotted. We decided to use 5% (v/v) IVTT expressed MKK1 and 30 minutes incubation (dotted line) prior to chymotrypsin digest for all future experiments. Datapoints denote the average of two technical repeats.

(C) End point measurement after 30 minutes incubation of 5% (v/v) ^{ca}MKK1 or 5% (v/v) ^{ca}MKK1$^{I9A/L11A}$ with the FRET sensor and ERK2 prior to additional 30 minutes incubation with chymotrypsin (derived from Figure A). Standard deviations derived from three biological repeats are shown, and as individual datapoints.

Source data are provided as a Source Data file.

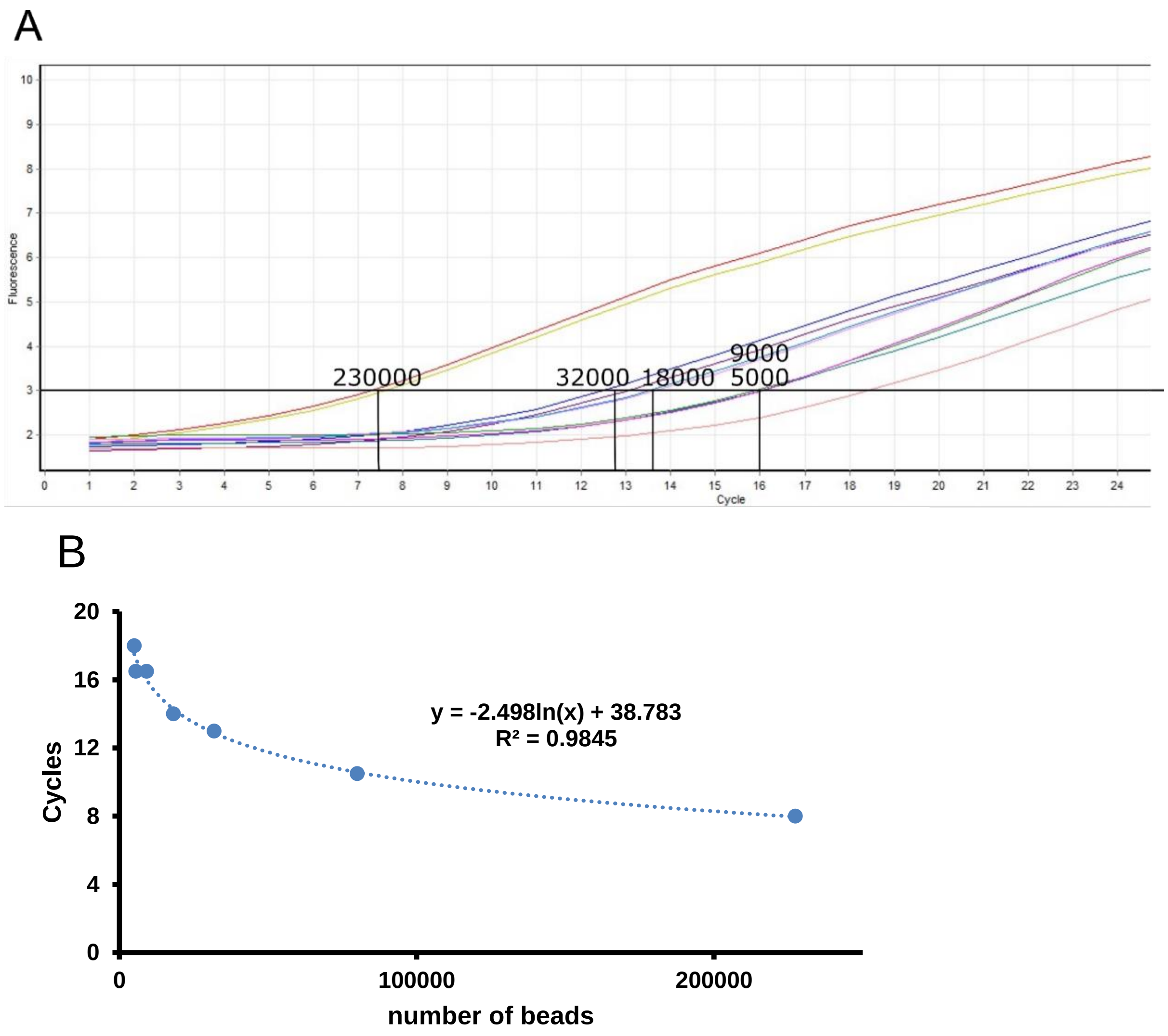

Supplementary Figure 9. Reducing bias of recovery by limited cycle PCR.

(A) Different numbers of *^{ca}MKK1* functionalised beads were tested in separate PCR reactions following DNA amplification by qPCR. For large amounts of template DNA (230,000 *MKK1*-functionalised beads), only a small number of cycles was required to get low-exponential amplification (black bar) (B) The number of cycles required to reach the low-exponential amplification threshold (Black bar – A) was exponentially correlated to the number of beads used as template molecules in the PCR reaction. We used this formula to calculate the number of cycles needed to recover sufficient quantities of D-domain DNA for beads of the low GFP gate (267,000 beads/PCR reaction – 8 cycles), medium GFP gate (35,000 beads/PCR reaction – 13 cycles) and high GFP gate (10,000 beads/PCR reaction – 16 cycles).

variant	gate	% of beads			Average % beads	st. dev.	seq counts	% seq counts
caMKK1	high	55	40	55	50.0	7.1	51	42%
	med	26	30	26	27.3	1.9	36	30%
	low	17	29	17	21.0	5.7	34	28%
caMKK1_I9A/L11A	high	4.5	2.8	7.2	4.8	1.8	0	0%
	med	9.6	5.7	14	9.8	3.4	6	23%
	low	85	91	78	84.7	5.3	20	77%

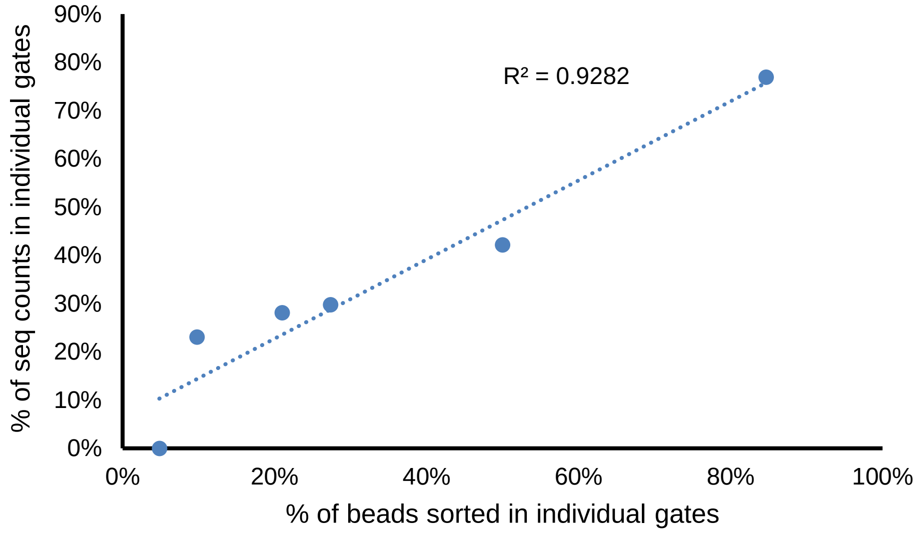

Supplementary Figure 10. Correlating the sorting events to the sequencing counts. The average percentage of beads sorted in the high, medium and low activity gates (for variants ^{ca}MKK1 and ^{ca}MKK1$^{I9A/L11A}$, in three parallel emulsions (displayed in **Supplementary Figure 7**) is well correlated (r^2= 0.92) with the percentage of sequencing counts of the two respective variants across the three different gates.

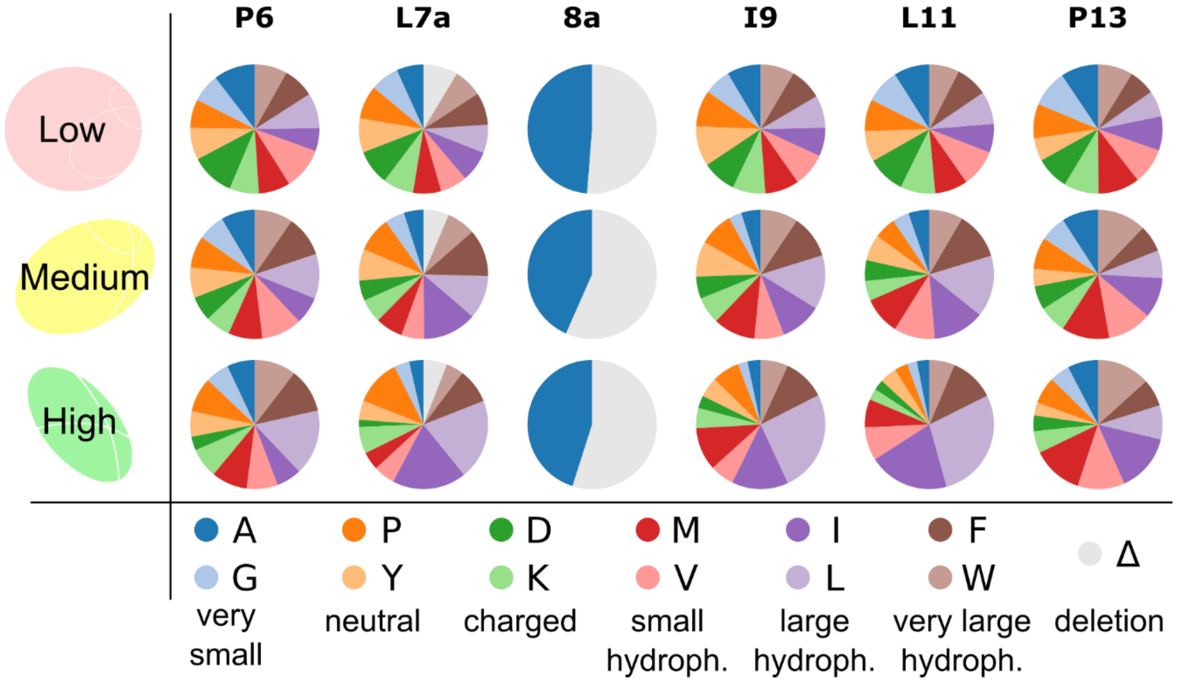

Supplementary Figure 11. The amino acid distribution of the unique D-domain sequences found in each gate. Raw data in **Supplementary Tables 1-3**.

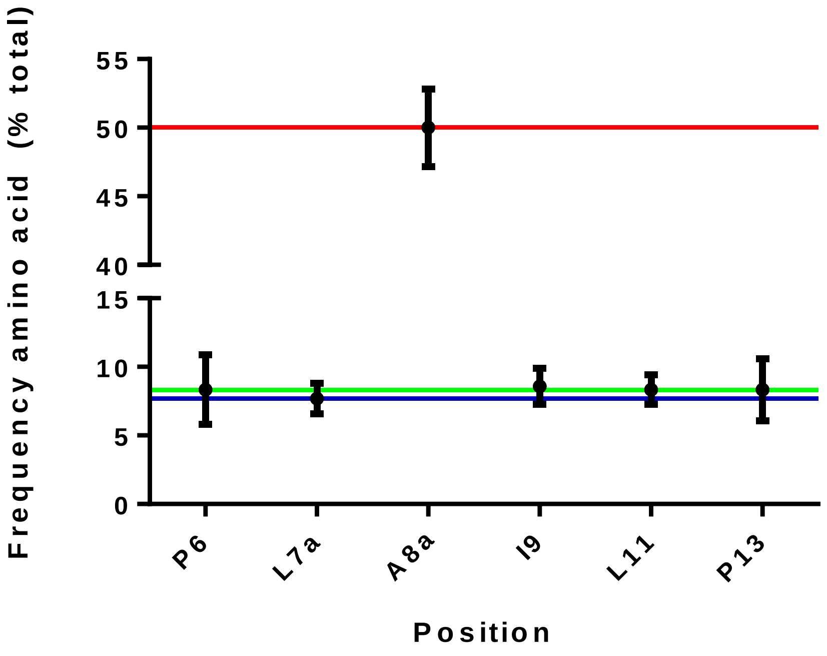

Supplementary Figure 12. The standard deviation and mean for the frequencies of each amino acid randomised at each position in low activity gate GFP sequences.

Because 85% of the sorted beads **(Supplementary Figure 7)** were sorted in the low activity gate, and 95.1% of unique sequences are found in said gate **(Figure 3A)**, the D-domain sequences recovered from the low activity gate at least once serve to confirm the homogeneity of the initial library which was not sequenced. To do so, the standard deviation of the amino acid distribution at each position **(Supplementary Table 3)** was calculated when compared to what is expected from a perfectly balanced starting library. For P6, I9, L11 and P13, the expected mean is 8.3%, shown as a green line (12 unique residues/position). For L7a the expected mean is 7.7%, shown as a blue line (12 residues or a single deletion). For A8a the predicted mean is 50%, shown as a red line (alanine or a single deletion).

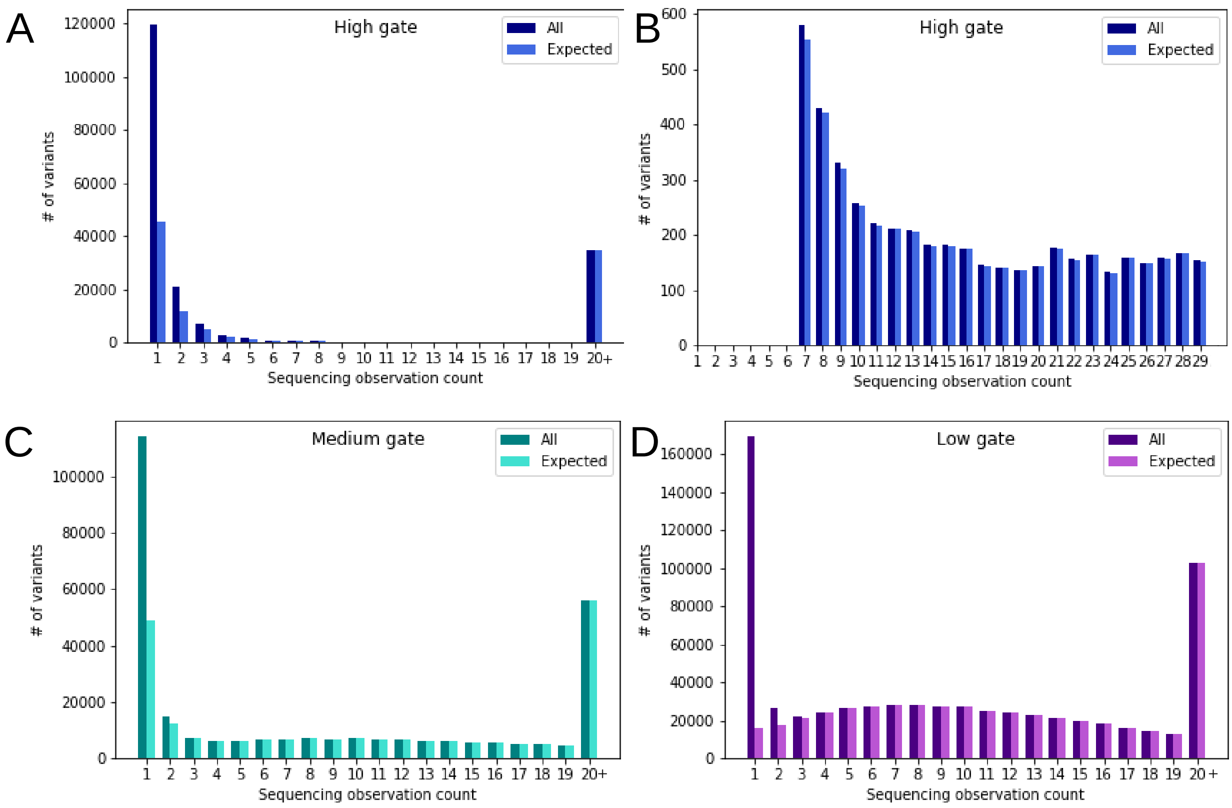

Supplementary Figure 13. The variation of the number of detected variants as a function of the number of reads.

A) The high gate is sequenced deeply, such that ~30K variants are detected 20 or more times (the rightmost column represents this sum). The variants that appear with only 1 or 2 supporting reads are disproportionately numerous (140K+) compared to the expected number of detected variants in the high gate (3.2% beads out of 3,500,000 beads (112,000 beads), which, through 7-fold oversampling, gives 16K variants). As the high gate is unlikely to sort all 7 beads for each variant considering the false negative rate (~50%, so that for each active variant 3.5 beads are expected on average in the high gate), the 112,000 beads sorted in the high activity gate are likely to encode (112,000/3.5) or 32K unique variants. Furthermore, only 1/3 of these variants fit with the expected mutations in the SpliMLiB libraries, which indicates that the low-abundance variants are primarily sequencing noise.

B) When choosing a high-gate cut-off between the likely true positive variants and sequencing noise, no clear cut-off is apparent. Any value above ~10 reads could be a reasonable if arbitrary cut-off.

C, D) The low and medium gates contain most of the library variants. Despite being sequenced with a higher total number of reads compared to the high gate, the large diversity in these gates results in a lower number of reads per variants. Both gates show a similar spike in erroneous variants that were only observed once, while variants that fit the expected mutation pattern predominate from 3-5 reads/variant onwards.

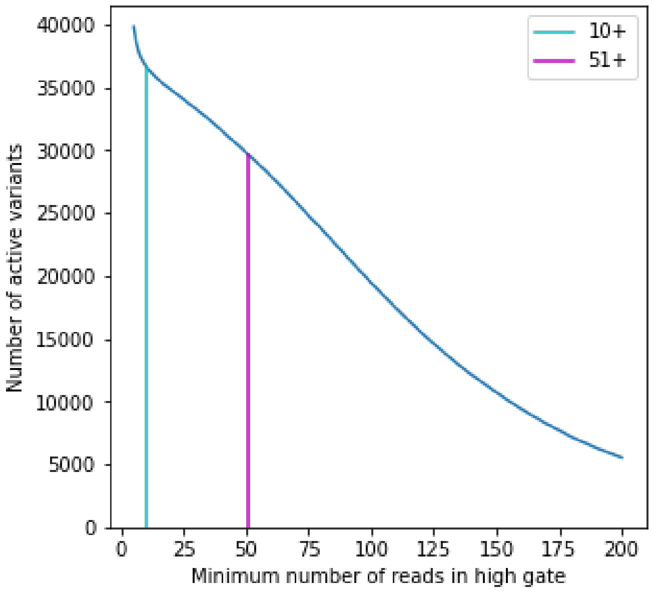

Supplementary Figure 14. The choice of the cutoff read count in the high gate (A,B) affects the number of variants included in the active dataset. The numbers for two possible choices (10+ and 51+, the latter being WT count) are demonstrated. The size of the active set as a function of sequencing count is smooth and shows no clear inflection point, making the exact choice flexible (see **Supplementary Note 2** for discussion)

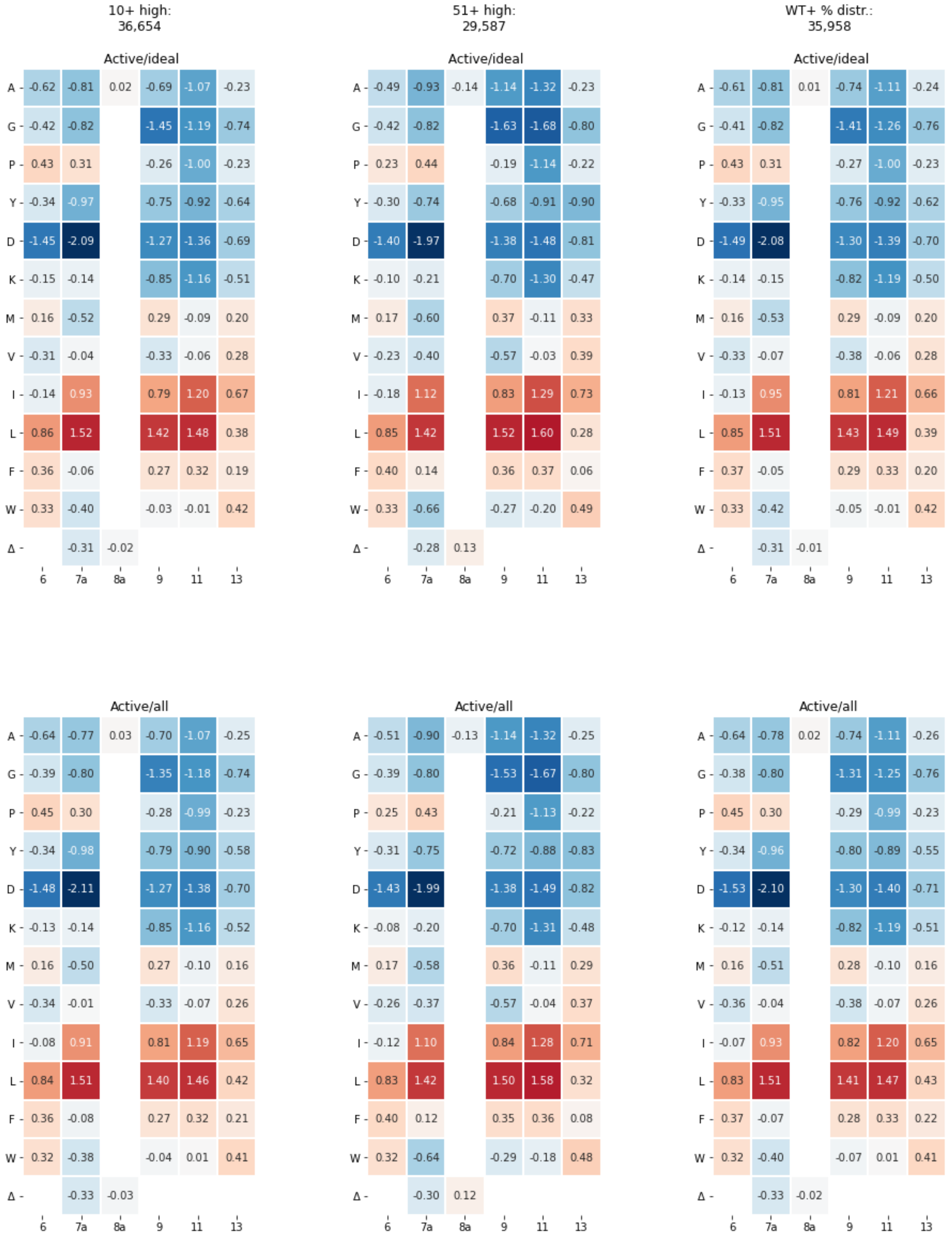

Supplementary Figure 15. Alternative formulations of the enrichment ratios yield identical results.

Here we show the single position enrichment heatmaps (as in **Figure 3B**, here reproduced in top row, middle column) calculated for alternative definitions of the enrichment ratios, with the numeric value of $\log_2 f^{active} / f^{ideal\ or\ all}$ superimposed. The top row charts show three possible choices of the active dataset, one more permissive (10+ reads in the high gate, not 51+) and one based on the distribution of reads across three sequencing gates (40+% reads in high gate, <30% reads in low gate). The bottom row charts use the same three options for the active dataset, but instead divide

the amino acid distribution in the active dataset by the amino acid distribution in all observed sequences.

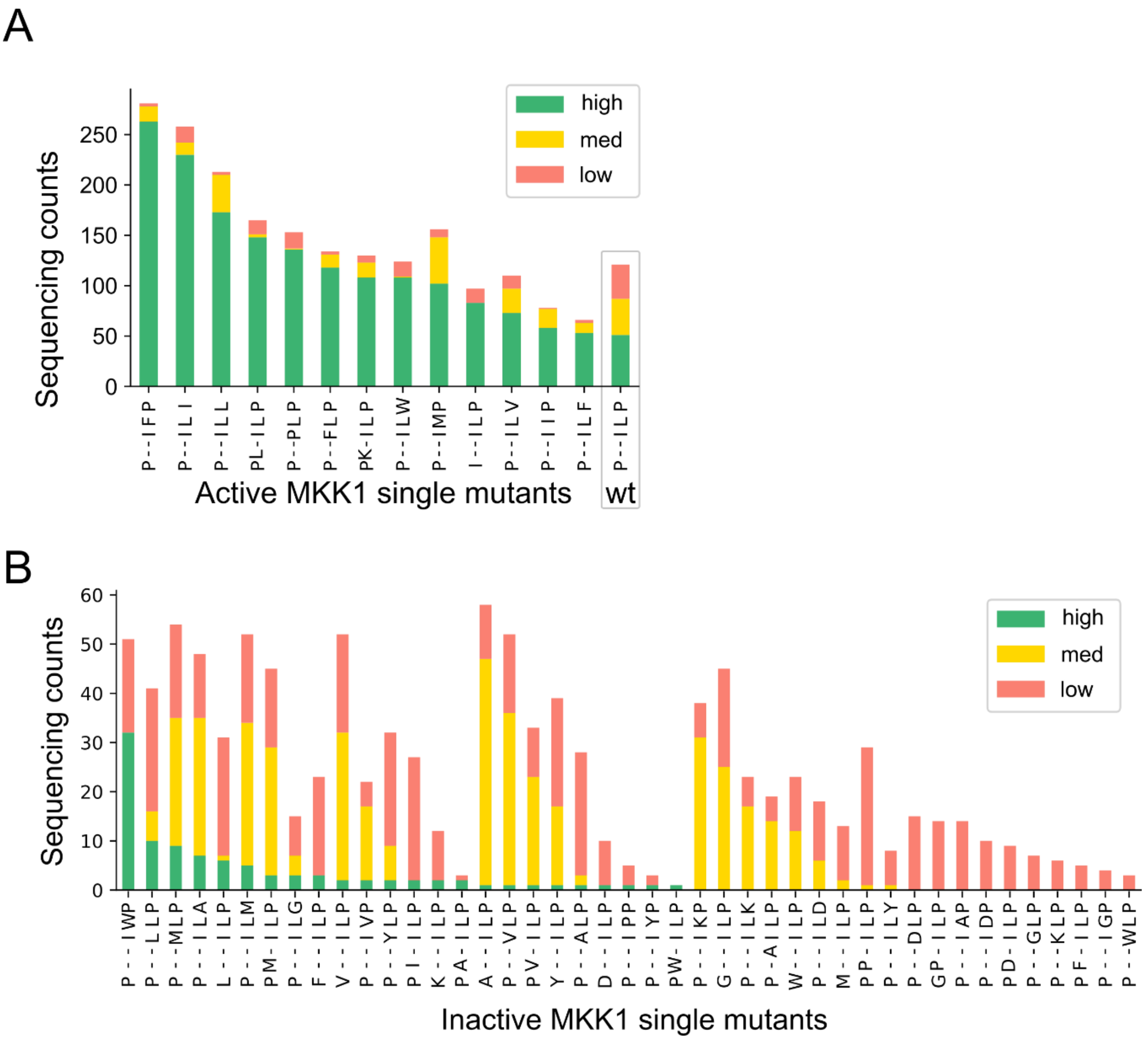

Supplementary Figure 16. The read distribution in MKK1 single mutants in the active variant set (A) and for less active/inactive variants (B).

The distinction between the active dataset and the remaining variants was set according to the sequencing read distribution of the WT, which therefore appears as the tail end of the active dataset (A). All other single amino acid mutants in the active dataset are more abundant in the high gate sequencing (green bar) and appear at a lower proportion in the low gate. (B) Of the variants in this plot all but one show a profile that clearly indicates reduced activity. The exception is the variant P--IWP, which may be borderline but was excluded; it does not have enough reads in the high gate (32<51), making functional assignment ambiguous – and thus falling outside the active variant dataset.

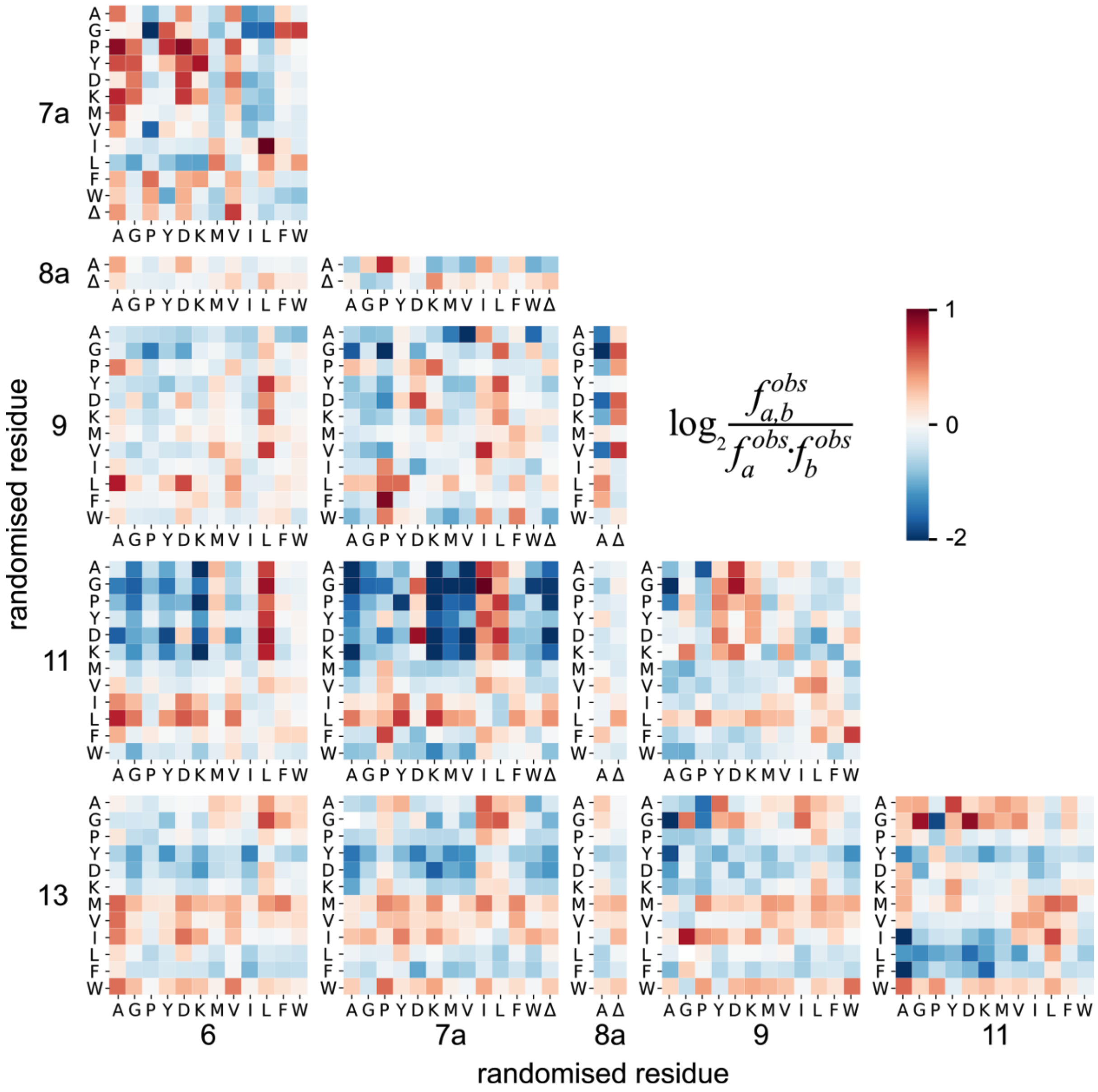

Supplementary Figure 17. A map of first-order epistasis in the D-domain shows Leu/Ile residues exhibit positive epistasis with non-preferred residues.

Each panel shows how the preference of a *pair* of amino acids in two positions compares to the expected frequency of that combination (e.g. for Leu at position 9 and a Tyr at position 11, the square is red, indicating that the combination is enriched). Since hydrophobic residues are enriched at all positions in the first place, pairs of hydrophobic residues are also likely to appear very frequently even in the absence of epistasis. This chart shows how the observed frequency of amino acid combinations deviates from the expectation: red colour indicates positive epistasis (more frequent than expected) and blue colour is negative epistasis (the combination is depleted from the dataset).

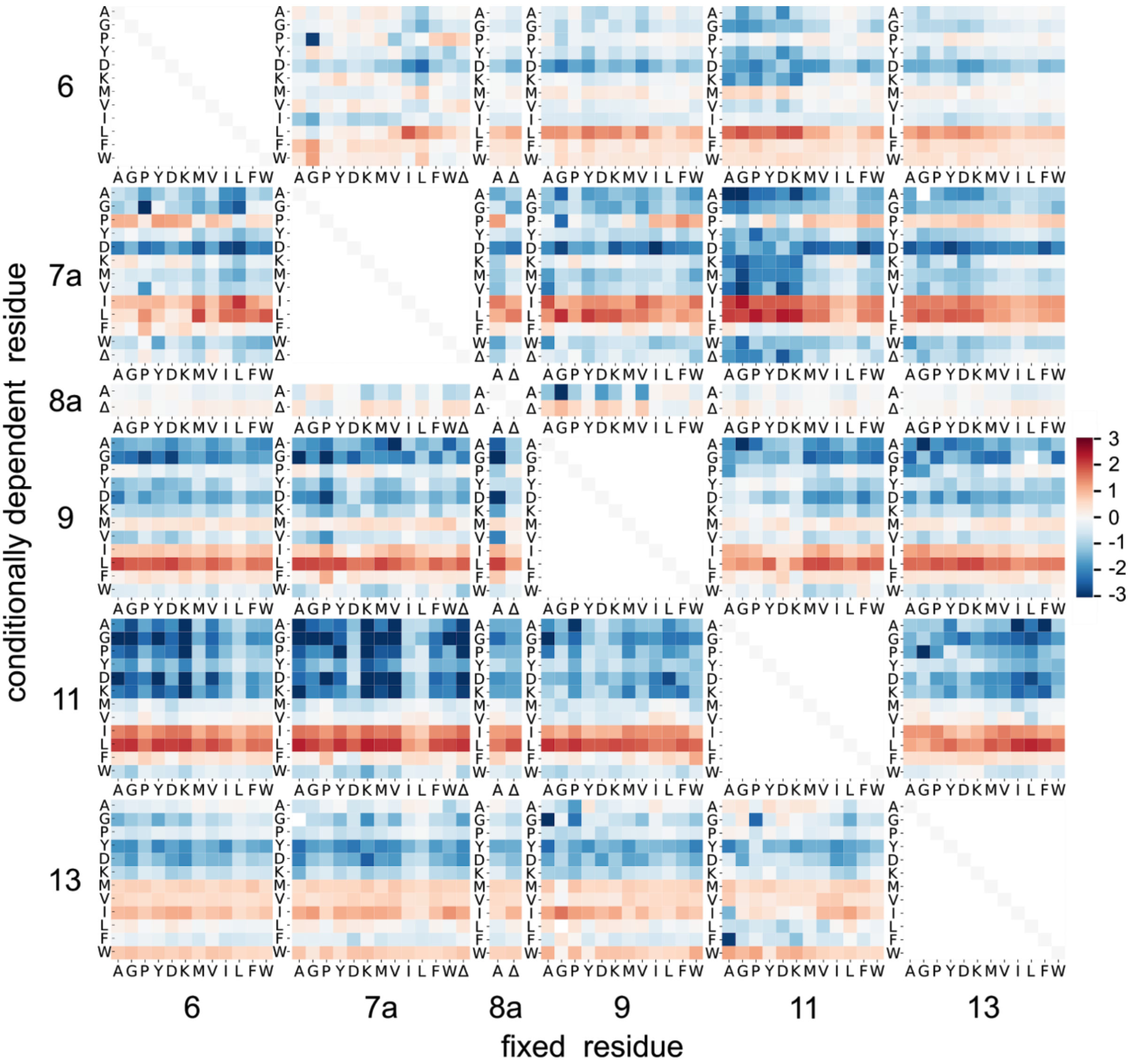

Supplementary Figure 18. An aid to detecting epistasis: a map of conditional probability*.*

Each square shows the relative preference for each amino acid in one position (each row, a) if a particular amino acid (each column, b) is fixed at the other position: $\frac{f_{a|b}^{obs}}{f_{a}^{id}}$. Consequently, the columns in each square are independent, but the amino acid probability in each column sums to 1. Note that the charts are not symmetrical across the diagonal.

As an example, consider the question “If there is a Met in position 6, what does that mean for sequence preferences at position 7a?”. This question is answered by the square for position 6 (first column, fixed) and position 7 (second row, variable). We fixed a Met in position 6, so look up the column for M: we see that a Leu is strongly preferred at position 7a.

Most combinations of randomised residues (panels in the figure) show a general ‘stripe of preference’ for large hydrophobic residues, especially Leu and Ile. In other words, the hydrophobic residues are preferred (y axis, conditional probability) regardless of which residues is present in the other position (x axis, fixed). However, on closer examination most of those ‘stripes’ change colour, indicating that the *magnitude* of the preference changes, i.e. the presence of epistasis. Additionally, some combinations

of positions and residues create pairs where the residue preferences switch: for example, the preferred residue in position 8a drastically depends on the residues in positions 7a and 9.

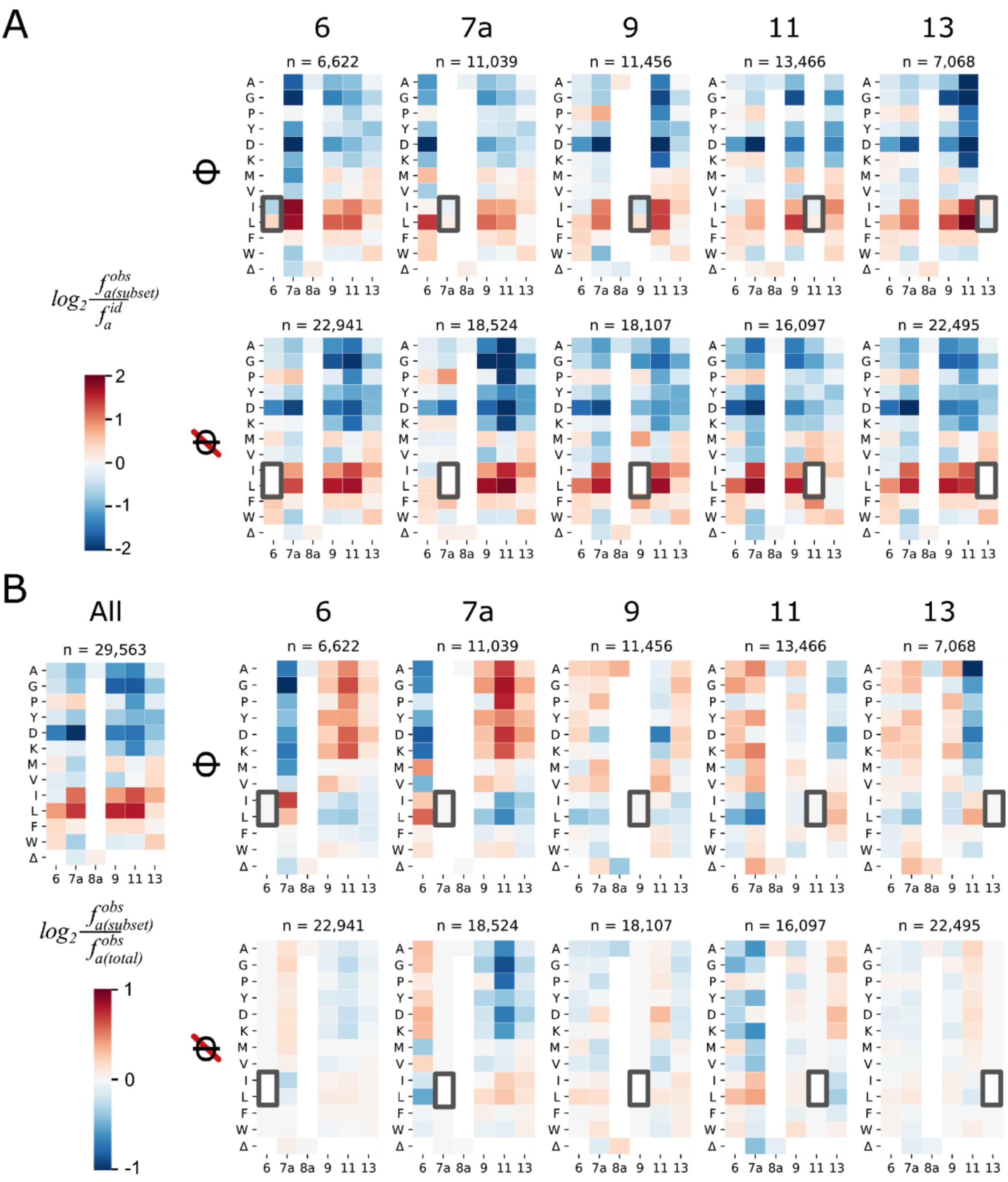

Supplementary Figure 19. A single Φ (Ile/Leu) residue is not sufficient for D-domain anchoring.

Heat maps for subsets of all active variants (29.563), where the data set is filtered for sequences that contain Φ or no Φ at the randomised positions, and mapping either amino acid enrichment (A) or epistasis (B).

(A). *Enrichment*. Top row: one position is restricted to Φ. Bottom row: The inverse restriction pattern, allowing only non-preferred residues at the chosen position. The presence of a single hydrophobic residue is not enough to 'relax' the amino acid in other positions, where Iso/Leu are still strongly enriched, and non-hydrophobic residues are depleted.

(B). *Epistasis*: Top left: The overall distribution in the full final active dataset, same as **Figure 3B.** The preferences in panel A are divided by the overall preferences in the full final active dataset, illustrating the change when certain positions are restricted to specific residues. Epistasis is mostly apparent in the subset of active sequences where 7a does not contain a Φ, where through negative epistasis the positions 9, 11 and 13 are unlikely to contain additional non-hydrophobic amino acids. This is especially true for position 11, where the presence of a non-hydrohobic residue disrupts the possibility of forming a **Φ-Φ** motif.

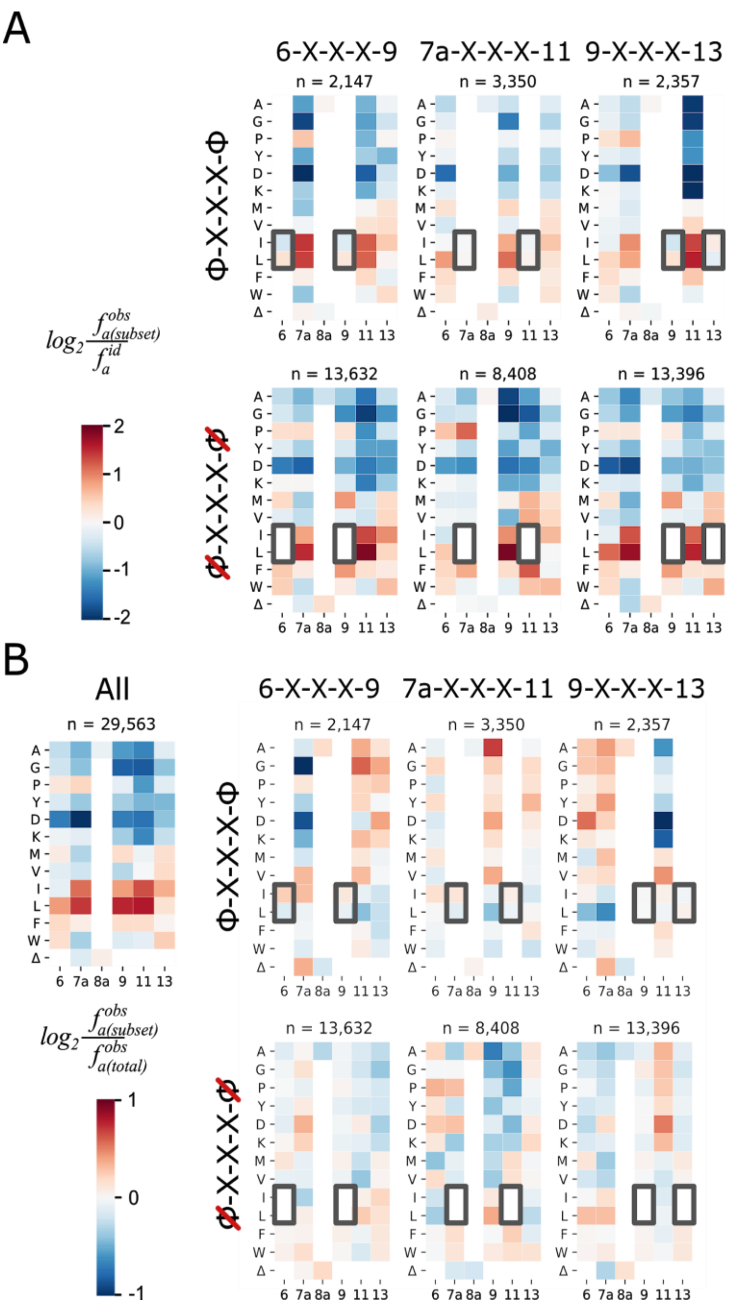

Supplementary Figure 20. Two non-adjacent Φ residues in a Φ-X-X-X-Φ motif are not sufficient.

As **Supplementary Figure 18**. Heat maps for subsets of all active variants (29.563), where the data set is filtered for sequences that contain a Φ-X-X-X-Φ motif or the negative complement where they contain no Φ at both positions.

(A). Enrichment If position 7a/11 or 9/13 are restricted to hydrophobic residues, then the residues in-between is also most likely to be hydrophobic – in this way, the previously described Φ-X-Φ motif is regenerated. In positions 6/9, the third hydrophobic residue may occupy either position 7a or 11, thus creating a two-residue motif with position 11. An interesting case occurs if neither 7a nor 11 contain a hydrophobic residue: then we see an intense enrichment of 9 Ile and recouping of hydrophobicity in positions 7a and 11.

(B). Epistasis is only present for specific amino acid pairs, and less prevalent than in the subsets where a Φ-X-Φ is present **(Figure 5B)**

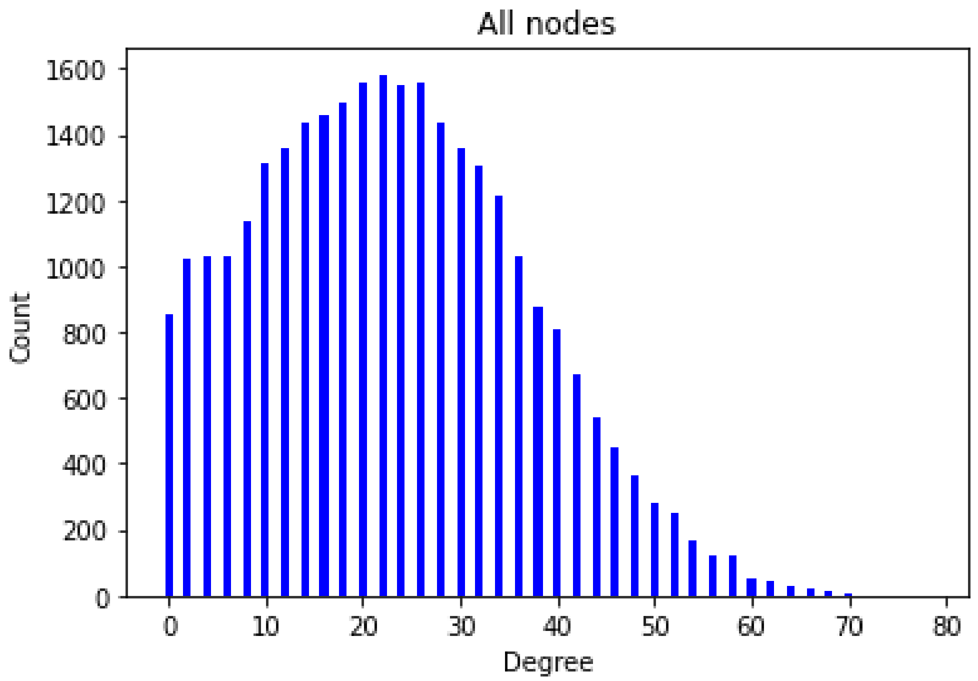

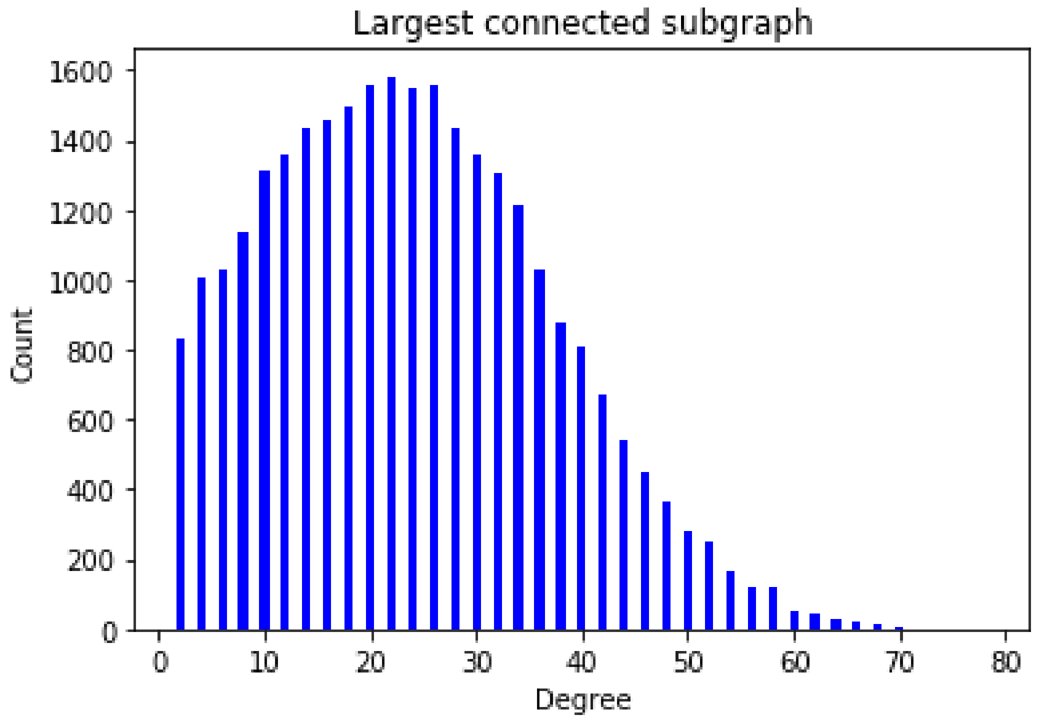

Supplementary Figure 21. The distribution of node degree in the full sequence similarity network and in the largest connected subgraph

The node degree is defined as the number of edges attached to a node; here it represents the number of variants in the active dataset that are accessible through a single amino acid mutation from a given node (variant). The distribution shows that the network is highly connected and that there is no sharp decrease in numbers at higher degree values.

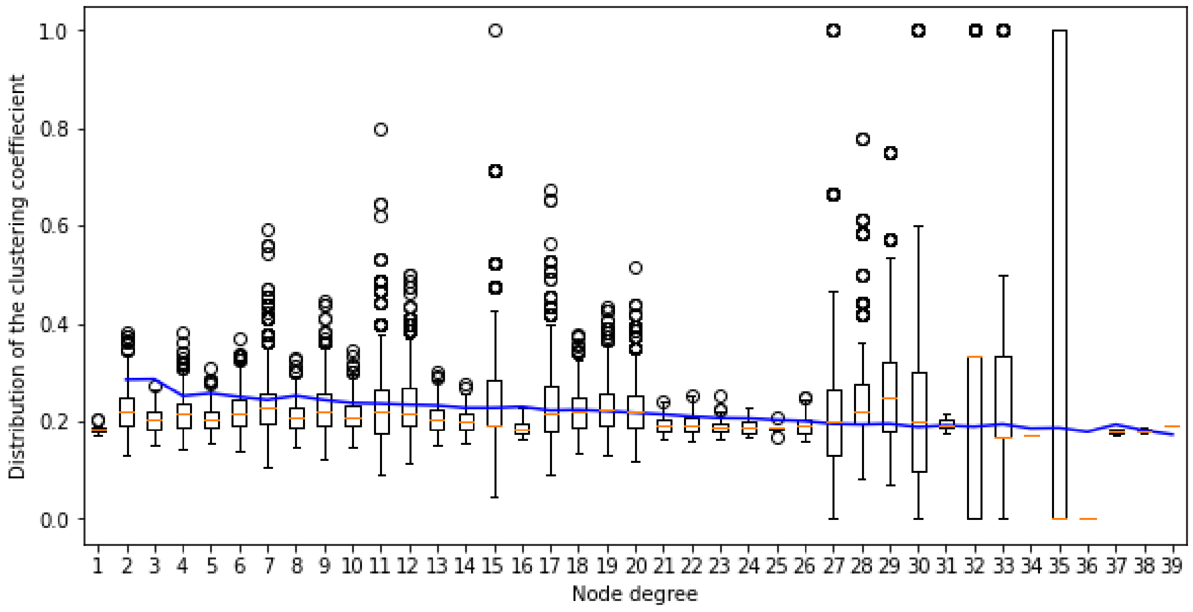

Supplementary Figure 22. The node clustering coefficient as a function of node degree in the sequence similarity network

The clustering coefficient measures the tendency of nodes in a graph to ‘cluster’ together, i.e. be all connected to each other. Specifically, if node A is connected to nodes B, C, D,… the coefficient of node A equals the number of edges connecting B, C, D… divided by the total number of edges that could connect them. Thus, the clustering coefficient can be interpreted as a probability of nodes connected to A also being connected to each other. Here, the clustering coefficient is calculated separately for all nodes in the largest connected subgraph (total number of nodes = 28,497) in the sequence similarity network of active D-domain variants, and plotted as a function of node degree.

The orange line in the middle of the box shows the median value and the box spans from first to third quartile range of the data. The whiskers extend to the last data point within 1.5×interquartile range past the box, showing the range of where most observations are found. Data point outside that range are plotted individually. The blue line shows the average node coefficient for each node degree. The trend of the clustering coefficient in this graph is fairly uniform across the graph, with a small number of outlier nodes with a very high clustering tendency.

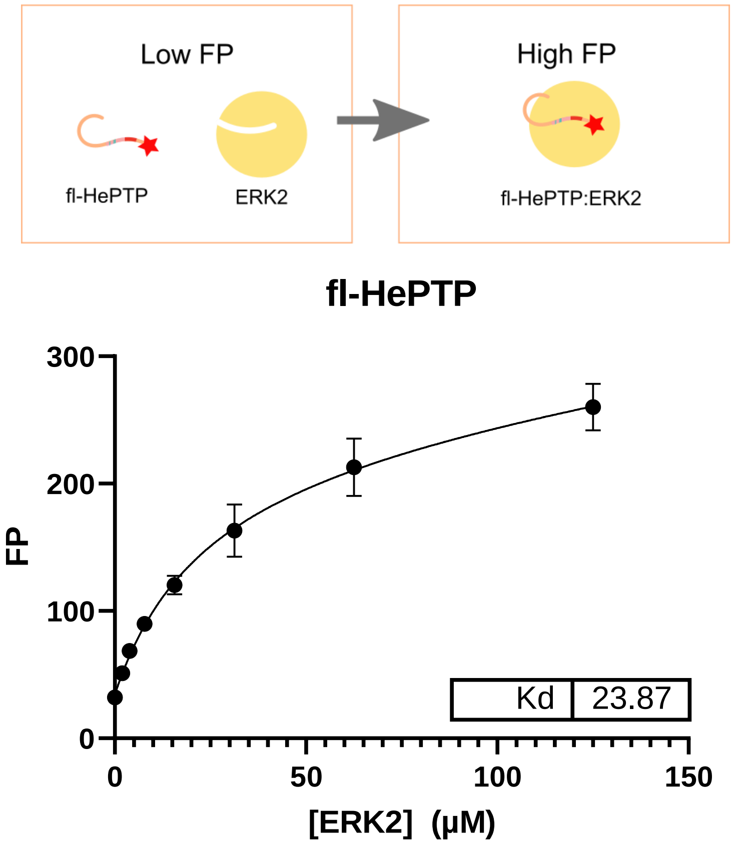

Supplementary Figure 23. Binding curves of ERK2 with fl-HePTP.

ERK2 was titrated against fl-HePTP as described, with the specific conditions described in the materials and methods and Garai et al.[4] ERK2 (dialysed to PBS_A) was concentrated and titrated against 40 nM fl-HePTP (in PBS_A). Data were fitted using GraphPad Prism 8.3 for Windows (GraphPad Software) according to the formula:

$$Y = Bmax * \frac{X}{(Kd + X)} + NS * X + Background$$

and gives a K_d of 23.87 ± 10 µM (Mean ± SEM). Standard Error derived from three biological repeats. Source data are provided as a Source Data file.

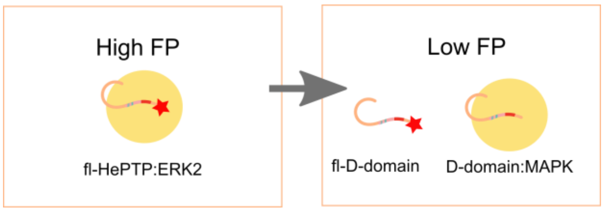

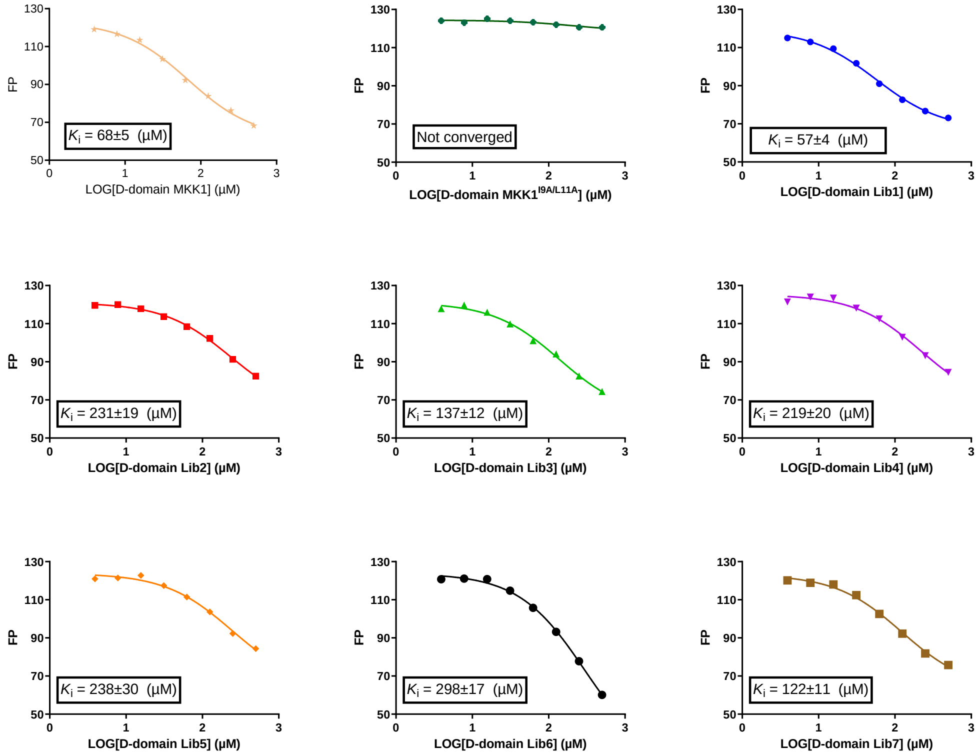

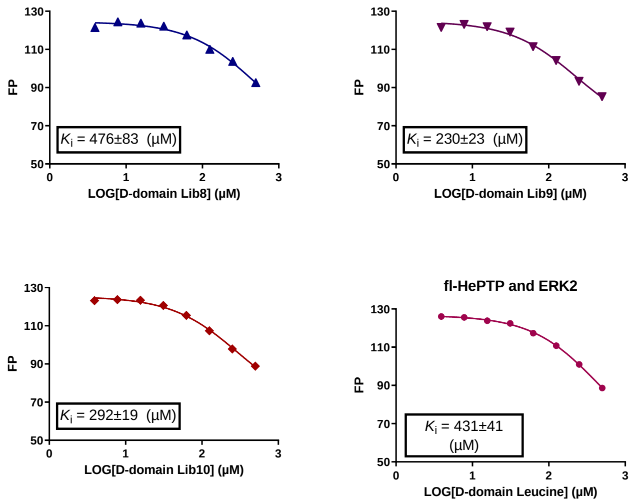

Name	Sequence	K_i (ERK2) (μM) [a,b]
Lib1	MPKKKLTIPALQFNM	57±4
Lib2	MPKKKFTPP-WQLNM	231±19
Lib3	MPKKKLTFP-LQINI	137±12
Lib4	MPKKKATIPALQLNI	219±20
Lib5	MPKKKWTIP-FQLNV	238±30
Lib6	MPKKKATPPALQVNW	298±17
Lib7	MPKKKLTIPAMQWNW	122±11
Lib8	MPKKKVTPPAFQFNM	476±83
Lib9	MPKKKATKP-LQLNW	230±34
Lib10	MPKKKATIPALQVNM	292±19
MKK1 I9A/L11A	MPKKKPT-P-AQANP	NC
MKK1	MPKKKPT-P-IQLNP	68±5
MKK6	SKGKKRNPG-LKIPK	65±6
MKK4	QGKRKALK--LNFAN	431±94
Leu-D	MPKKKLTLP-LQLNI	431±41

[a]NC = Not converged [b]Binding affinities are shown with their SE

Supplementary Figure 24. Competition assay between non-fluorescent D-domains and ERK2 incubated with fl-HePTP.

ERK2 (25 μM) was complexed with 40 nM fl-D-domain before titrating non-fluorescent D-domain against the complex as described.[4] Data points are averaged from two technical replicates. Source data are provided as a Source Data file.

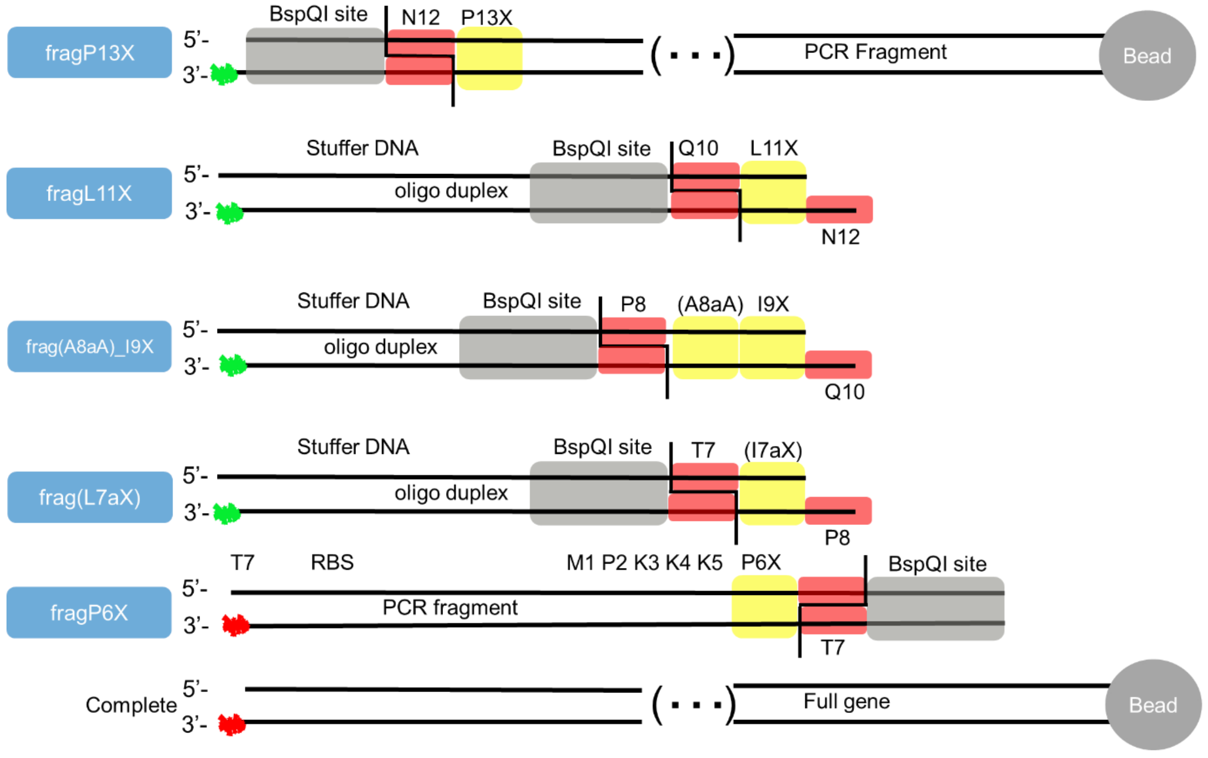

Supplementary Figure 25. SPliMLiB design.

A detailed procedure on SPliMLiB library assembly has been previously described.[2] The genes were assembled with the "stop codon-end" attached to the bead, working in reverse, so that the last fragment to be added contained the promotor. This way, only fully assembled genes will contain the promotor sequence necessary for expression. All oligonucleotides required for assembly are depicted in **Supplementary Table 6**. FragP13X is prepared via PCR. Use of a DBCO-functionalised reverse primer (T7T_DBCO) for all P13X fragments allows for a click reaction of the first fragment on beads (in parallel, split reactions). FragL11X, frag(A8aA)_I9X and frag(L7aX) were synthesised (Sigma) as single-strand oligonucleotides. The phosphorylated and duplexed product for these fragments is shown. fragP6x was again prepared via PCR, this time with a Cy5 functionalised LMB forward primer for all fragments. Beads which were functionalised in parallel with fragP13X DNA fragments were pooled, and the immobilised DNA was digested with BspQI. The beads are split, and each unique fragL11X was ligated in parallel. The beads were again pooled and the cycle is repeated. DNA fragments belonging to fragP6X were digested with BspQI before being ligated on in the last split. By splitting the beads before each ligation step, monoclonality of the beads is guaranteed. The beads are mixed in each digestion step, so all possible combinations of site saturated mutations are represented in the library on beads. The theoretical library size is 539,136 (12*13*2*12*12*12 (P6X*L7X*A8X*I9*L11X*P13X)).

Supplementary Table 1. Amino acid distribution of high gate recovered D-domains.

Raw number (top) of variants in the high gate that appear 10 or more times, with randomised amino acid counts at each position (4.5 million counts total). The lower table shows the same data expressed as % of all variants considered in this analysis and is used for graphical representation in **Supplementary Figure 11.**

**High (A)**	**6**	**7a**	**8a**	**9**	**11**	**13**
**A**	318033	162796	2045200	151025	143375	348236
**D**	156812	80272	0	143520	119528	178984
**F**	503808	396898	0	483081	511157	310544
**G**	269975	173540	0	108944	110428	227625
**I**	286203	843938	0	643111	918900	661938
**K**	339340	305212	0	228953	129386	248349
**L**	745630	913516	0	1160722	1272684	387858
**M**	407766	206904	0	489773	318561	578141
**P**	395032	525780	0	315651	150232	308007
**V**	353316	242767	0	273265	379452	534812
**W**	470755	198002	0	310149	287990	603606
**Y**	289297	218236	0	227773	194274	147867
**Δ**	0	268106	2490767	0	0	0

**High (%)**	**6**	**7a**	**8a**	**9**	**11**	**13**
**A**	7.0%	3.6%	45.1%	3.3%	3.2%	7.7%
**D**	3.5%	1.8%	0.0%	3.2%	2.6%	3.9%
**F**	11.1%	8.8%	0.0%	10.7%	11.3%	6.8%
**G**	6.0%	3.8%	0.0%	2.4%	2.4%	5.0%
**I**	6.3%	18.6%	0.0%	14.2%	20.3%	14.6%
**K**	7.5%	6.7%	0.0%	5.0%	2.9%	5.5%
**L**	16.4%	20.1%	0.0%	25.6%	28.1%	8.6%
**M**	9.0%	4.6%	0.0%	10.8%	7.0%	12.7%
**P**	8.7%	11.6%	0.0%	7.0%	3.3%	6.8%
**V**	7.8%	5.4%	0.0%	6.0%	8.4%	11.8%
**W**	10.4%	4.4%	0.0%	6.8%	6.3%	13.3%
**Y**	6.4%	4.8%	0.0%	5.0%	4.3%	3.3%
**Δ**	0.0%	5.9%	54.9%	0.0%	0.0%	0.0%

Supplementary Table 2. Amino acid distribution of medium gate recovered D-domains.

Raw number (top) of variants in the Medium gate that appear 3 or more times, with randomised amino acid counts at each position (2.9 million counts total). The lower table shows the same data expressed as % of all variants considered in this analysis and is used for graphical representation in **Supplementary Figure 11.**

**Med**	**6**	**7a**	**8a**	**9**	**11**	**13**
**A**	248133	146731	1285190	147755	156452	267164
**D**	180242	147406	0	163187	153534	176399
**F**	300720	344881	0	315105	347191	199896
**G**	199498	146418	0	98666	125538	191899
**I**	204506	392651	0	302866	377069	296219
**K**	185234	176460	0	196744	146802	201529
**L**	328181	319317	0	394712	448815	207928
**M**	250686	203460	0	308064	277147	347002
**P**	235684	255692	0	249962	161190	234226
**V**	295608	170975	0	220565	299249	323835
**W**	280859	212373	0	278040	248167	355993
**Y**	223970	229383	0	257655	192167	131231
**Δ**	0	187574	1648131	0	0	0

**Med (%)**	**6**	**7a**	**8a**	**9**	**11**	**13**
**A**	8.5%	5.0%	43.8%	5.0%	5.3%	9.1%
**D**	6.1%	5.0%	0.0%	5.6%	5.2%	6.0%
**F**	10.3%	11.8%	0.0%	10.7%	11.8%	6.8%
**G**	6.8%	5.0%	0.0%	3.4%	4.3%	6.5%
**I**	7.0%	13.4%	0.0%	10.3%	12.9%	10.1%
**K**	6.3%	6.0%	0.0%	6.7%	5.0%	6.9%
**L**	11.2%	10.9%	0.0%	13.5%	15.3%	7.1%
**M**	8.5%	6.9%	0.0%	10.5%	9.4%	11.8%
**P**	8.0%	8.7%	0.0%	8.5%	5.5%	8.0%
**V**	10.1%	5.8%	0.0%	7.5%	10.2%	11.0%
**W**	9.6%	7.2%	0.0%	9.5%	8.5%	12.1%
**Y**	7.6%	7.8%	0.0%	8.8%	6.6%	4.5%
**Δ**	0.0%	6.4%	56.2%	0.0%	0.0%	0.0%

Supplementary Table 3. Amino acid distribution of low gate recovered D-domains.

Raw number (top) of variants in the Low gate that appear 3 or more times, with randomised amino acid counts at each position (6.6 million counts total). The lower table shows the same data expressed as % of all variants considered in this analysis and is used for graphical representation in **Supplementary Figure 11.**

**Low**	**6**	**7a**	**8a**	**9**	**11**	**13**
**A**	695070	449801	3244833	566308	598577	627045
**D**	725912	586390	0	568929	632682	557254
**F**	517451	538600	0	537625	532723	434371
**G**	472230	478143	0	446286	569709	619266
**I**	385817	519925	0	475616	480719	571516
**K**	487957	512146	0	546141	580844	576921
**L**	585709	473006	0	539650	544418	434473
**M**	529132	467769	0	569000	547852	695607
**P**	479386	558505	0	605234	532670	571385
**V**	699899	449026	0	565227	624031	602944
**W**	542932	476249	0	562738	499163	581199
**Y**	532072	569658	0	670813	510179	381586
**Δ**	0	574349	3408734	0	0	0

**Low (%)**	**6**	**7a**	**8a**	**9**	**11**	**13**
**A**	10.4%	6.8%	48.8%	8.5%	9.0%	9.4%
**D**	10.9%	8.8%	0.0%	8.6%	9.5%	8.4%
**F**	7.8%	8.1%	0.0%	8.1%	8.0%	6.5%
**G**	7.1%	7.2%	0.0%	6.7%	8.6%	9.3%
**I**	5.8%	7.8%	0.0%	7.1%	7.2%	8.6%
**K**	7.3%	7.7%	0.0%	8.2%	8.7%	8.7%
**L**	8.8%	7.1%	0.0%	8.1%	8.2%	6.5%
**M**	8.0%	7.0%	0.0%	8.6%	8.2%	10.5%
**P**	7.2%	8.4%	0.0%	9.1%	8.0%	8.6%
**V**	10.5%	6.7%	0.0%	8.5%	9.4%	9.1%
**W**	8.2%	7.2%	0.0%	8.5%	7.5%	8.7%
**Y**	8.0%	8.6%	0.0%	10.1%	7.7%	5.7%
**Δ**	0.0%	8.6%	51.2%	0.0%	0.0%	0.0%

Supplementary Table 4. Protein-encoding DNA sequences and their translation for all proteins used in this study.

MKK1 – UniProt ID Q02750 (Part of pIVEX2.3d plasmid)

DNA Sequence

ATGCCCAAGAAGAAGCCGACGCCCATCCAGCTGAACCCGGCCCCCGACGGCTCTGCAGTTAACGGGAC
CAGCTCTGCGGAGACCAACTTGGAGGCCTTGCAGAAGAAGCTGGAGGAGCTAGAGCTTGATGAGCAGC
AGCGAAAGCGCCTTGAGGCCTTTCTTACCCAGAAGCAGAAGGTGGGAGAACTGAAGGATGACGACTTTG
AGAAGATCAGTGAGCTGGGGGCTGGCAATGGCGGTGTGGTGTTCAAGGTCTCCCACAAGCCTTCTGGCC
TGGTCATGGCCAGAAAGCTAATTCATCTGGAGATCAAACCCGCAATCCGGAACCAGATCATAAGGGAGCT
GCAGGTTCTGCATGAGTGCAACTCTCCGTACATCGTGGGCTTCTATGGTGCGTTCTACAGCGATGGCGA
GATCAGTATCTGCATGGAGCACATGGATGGAGGTTCTCTGGATCAAGTCCTGAAGAAAGCTGGAAGAATT
CCTGAACAAATTTTAGGAAAAGTTAGCATTGCTGTAATAAAAGGCCTGACATATCTGAGGGAGAAGCACAA
GATCATGCACAGAGATGTCAAGCCCTCCAACATCCTAGTCAACTCCCGTGGGGAGATCAAGCTCTGTGAC
TTTGGGGTCAGCGGGCAGCTCATCGACTCCATGGCCAACTCCTTCGTGGGCACAAGGTCCTACATGTCG
CCAGAAAGACTCCAGGGGACTCATTACTCTGTGCAGTCAGACATCTGGAGCATGGGACTGTCTCTGGTA
GAGATGGCGGTTGGGAGGTATCCCATCCCTCCTCCAGATGCCAAGGAGCTGGAGCTGATGTTTGGGTGC
CAGGTGGAAGGAGATGCGGCTGAGACCCCACCCAGGCCAAGGACCCCCGGGAGGCCCCTTAGCTCATA
CGGAATGGACAGCCGACCTCCCATGGCAATTTTTGAGTTGTTGGATTACATAGTCAACGAGCCTCCTCCA
AAACTGCCCAGTGGAGTGTTCAGTCTGGAATTTCAAGATTTTGTGAATAAATGCTTAATAAAAAACCCCGC
AGAGAGAGCAGATTTGAAGCAACTCATGGTTCATGCTTTTATCAAGAGATCTGATGCTGAGGAAGTGGAT
TTTGCAGGTTGGCTCTGCTCCACCATCGGCCTTAACCAGCCCAGCACACCAACCCATGCTGCTGGCGTC
GGATCCACTAGTGGTTATCCGTATGATGTACCAGATTATGCAAGCCTAACTAGTTAG

Translation

MPKKKPTPIQLNPAPDGSAVNGTSSAETNLEALQKKLEELELDEQQRKRLEAFLTQKQKVGELKDDDFEKISEL
GAGNGGVVFKVSHKPSGLVMARKLIHLEIKPAIRNQIIRELQVLHECNSPYIVGFYGAFYSDGEISICMEHMDGG
SLDQVLKKAGRIPEQILGKVSIAVIKGLTYLREKHKIMHRDVKPSNILVNSRGEIKLCDFGVSGQLIDSMANSFVG
TRSYMSPERLQGTHYSVQSDIWSMGLSLVEMAVGRYPIPPPDAKELELMFGCQVEGDAAETPPRPRTPGRPL
SSYGMDSRPPMAIFELLDYIVNEPPPKLPSGVFSLEFQDFVNKCLIKNPAERADLKQLMVHAFIKRSDAEEVDF
AGWLCSTIGLNQPSTPTHAAGVGSTSGYPYDVPDYASLTS-

MKK1HA-tag

MKK1_S218D_S222D (^{ca}MKK1$^{S218D/S222D}$) (Part of pIVEX2.3d plasmid).[5]

DNA sequence

ATGCCCAAGAAGAAGCCGACGCCCATCCAGCTGAACCCGGCCCCCGACGGCTCTGCAGTTAACGGGAC
CAGCTCTGCGGAGACCAACTTGGAGGCCTTGCAGAAGAAGCTGGAGGAGCTAGAGCTTGATGAGCAGC
AGCGAAAGCGCCTTGAGGCCTTTCTTACCCAGAAGCAGAAGGTGGGAGAACTGAAGGATGACGACTTTG
AGAAGATCAGTGAGCTGGGGGCTGGCAATGGCGGTGTGGTGTTCAAGGTCTCCCACAAGCCTTCTGGCC
TGGTCATGGCCAGAAAGCTAATTCATCTGGAGATCAAACCCGCAATCCGGAACCAGATCATAAGGGAGCT
GCAGGTTCTGCATGAGTGCAACTCTCCGTACATCGTGGGCTTCTATGGTGCGTTCTACAGCGATGGCGA
GATCAGTATCTGCATGGAGCACATGGATGGAGGTTCTCTGGATCAAGTCCTGAAGAAAGCTGGAAGAATT
CCTGAACAAATTTTAGGAAAAGTTAGCATTGCTGTAATAAAAGGCCTGACATATCTGAGGGAGAAGCACAA
GATCATGCACAGAGATGTCAAGCCCTCCAACATCCTAGTCAACTCCCGTGGGGAGATCAAGCTCTGTGAC
TTTGGGGTCAGCGGGCAGCTCATCGACGACATGGCCAACGACTTCGTGGGCACAAGGTCCTACATGTCG
CCAGAAAGACTCCAGGGGACTCATTACTCTGTGCAGTCAGACATCTGGAGCATGGGACTGTCTCTGGTA
GAGATGGCGGTTGGGAGGTATCCCATCCCTCCTCCAGATGCCAAGGAGCTGGAGCTGATGTTTGGGTGC
CAGGTGGAAGGAGATGCGGCTGAGACCCCACCCAGGCCAAGGACCCCCGGGAGGCCCCTTAGCTCATA
CGGAATGGACAGCCGACCTCCCATGGCAATTTTTGAGTTGTTGGATTACATAGTCAACGAGCCTCCTCCA
AAACTGCCCAGTGGAGTGTTCAGTCTGGAATTTCAAGATTTTGTGAATAAATGCTTAATAAAAAACCCCGC
AGAGAGAGCAGATTTGAAGCAACTCATGGTTCATGCTTTTATCAAGAGATCTGATGCTGAGGAAGTGGAT
TTTGCAGGTTGGCTCTGCTCCACCATCGGCCTTAACCAGCCCAGCACACCAACCCATGCTGCTGGCGTC
GGATCCACTAGTGGTTATCCGTATGATGTACCAGATTATGCAAGCCTAACTAGTTAG

Translation

MPKKKPTPIQLNPAPDGSAVNGTSSAETNLEALQKKLEELELDEQQRKRLEAFLTQKQKVGELKDDDFEKISEL
GAGNGGVVFKVSHKPSGLVMARKLIHLEIKPAIRNQIIRELQVLHECNSPYIVGFYGAFYSDGEISICMEHMDGG
SLDQVLKKAGRIPEQILGKVSIAVIKGLTYLREKHKIMHRDVKPSNILVNSRGEIKLCDFGVSGQLIDDMANDFVG
TRSYMSPERLQGTHYSVQSDIWSMGLSLVEMAVGRYPIPPPDAKELELMFGCQVEGDAAETPPRPRTPGRPL
SSYGMDSRPPMAIFELLDYIVNEPPPKLPSGVFSLEFQDFVNKCLIKNPAERADLKQLMVHAFIKRSDAEEVDF
AGWLCSTIGLNQPSTPTHAAGVGSTSGYPYDVPDYASLTS-

MKK1S218D_S222DHA-tag

MKK1_Δ43-51_S218D_M219D_N221D_S222D* (caMKK1) (Part of pIVEX2.3d plasmid).[5]
*Numbering of mutants is based on the wildtype MKK1 sequence without the deletion.

DNA sequence
ATGCCCAAGAAGAAGCCGACGCCCATCCAGCTGAACCCGGCCCCCGACGGCTCTGCAGTTAACGGGAC
CAGCTCTGCGGAGACCAACTTGGAGGCCTTGCAGAAGAAGCTGGAGGAGCTAGAGCTTGATGCCTTTCT
TACCCAGAAGCAGAAGGTGGGAGAACTGAAGGATGACGACTTTGAGAAGATCAGTGAGCTGGGGGCTG
GCAATGGCGGTGTGGTGTTCAAGGTCTCCCACAAGCCTTCTGGCCTGGTCATGGCCAGAAAGCTAATTC
ATCTGGAGATCAAACCCGCAATCCGGAACCAGATCATAAGGGAGCTGCAGGTTCTGCATGAGTGCAACT
CTCCGTACATCGTGGGCTTCTATGGTGCGTTCTACAGCGATGGCGAGATCAGTATCTGCATGGAGCACAT
GGATGGAGGTTCTCTGGATCAAGTCCTGAAGAAAGCTGGAAGAATTCCTGAACAAATTTTAGGAAAAGTT
AGCATTGCTGTAATAAAAGGCCTGACATATCTGAGGGAGAAGCACAAGATCATGCACAGAGATGTCAAGC
CCTCCAACATCCTAGTCAACTCCCGTGGGGAGATCAAGCTCTGTGACTTTGGGGTCAGCGGGCAGCTCA
TCGACGATGATGCCGACGACTTCGTGGGCACAAGGTCCTACATGTCGCCAGAAAGACTCCAGGGGACTC
ATTACTCTGTGCAGTCAGACATCTGGAGCATGGGACTGTCTCTGGTAGAGATGGCGGTTGGGAGGTATC
CCATCCCTCCTCCAGATGCCAAGGAGCTGGAGCTGATGTTTGGGTGCCAGGTGGAAGGAGATGCGGCT
GAGACCCCACCCAGGCCAAGGACCCCCGGGAGGCCCCTTAGCTCATACGGAATGGACAGCCGACCTCC
CATGGCAATTTTTGAGTTGTTGGATTACATAGTCAACGAGCCTCCTCCAAAACTGCCCAGTGGAGTGTTCA
GTCTGGAATTTCAAGATTTTGTGAATAAATGCTTAATAAAAAACCCCGCAGAGAGAGCAGATTTGAAGCAA
CTCATGGTTCATGCTTTTATCAAGAGATCTGATGCTGAGGAAGTGGATTTTGCAGGTTGGCTCTGCTCCAC
CATCGGCCTTAACCAGCCCAGCACACCAACCCATGCTGCTGGCGTCGGATCCACTAGTGGTTATCCGTA
TGATGTACCAGATTATGCAAGCCTAACTAGTTAG

Translation
MPKKKPTPIQLNPAPDGSAVNGTSSAETNLEALQKKLEELELDΔΔΔΔΔΔΔΔAFLTQKQKVGELKDDDFEKISEL
GAGNGGVVFKVSHKPSGLVMARKLIHLEIKPAIRNQIIRELQVLHECNSPYIVGFYGAFYSDGEISICMEHMDGG
SLDQVLKKAGRIPEQILGKVSIAVIKGLTYLREKHKIMHRDVKPSNILVNSRGEIKLCDFGVSGQLIDDDADDFVG
TRSYMSPERLQGTHYSVQSDIWSMGLSLVEMAVGRYPIPPPDAKELELMFGCQVEGDAAETPPRPRTPGRPL
SSYGMDSRPPMAIFELLDYIVNEPPPKLPSGVFSLEFQDFVNKCLIKNPAERADLKQLMVHAFIKRSDAEEVDF
AGWLCSTIGLNQPSTPTHAAGVGSTSGYPYDVPDYASLTS-

MKK1 Δ43-51, S218D_M219D_N221D_S222D HA-tag

MKK1_I9A_L11A_Δ43-51_S218D_M219D_N221D_S222D* (caMKK1I9A/L11A) (Part of pIVEX2.3d plasmid).[5,6]
*Numbering of mutants is based on the wildtype MKK1 sequence without the deletion.

DNA sequence
ATGCCCAAGAAGAAGCCGACGCCCGCGCAAGCTAACCCGGCCCCCGACGGCTCTGCAGTTAACGGGAC
CAGCTCTGCGGAGACCAACTTGGAGGCCTTGCAGAAGAAGCTGGAGGAGCTAGAGCTTGATGAGCAGC
AGCGAAAGCGCCTTGAGGCCTTTCTTACCCAGAAGCAGAAGGTGGGAGAACTGAAGGATGACGACTTTG
AGAAGATCAGTGAGCTGGGGGCTGGCAATGGCGGTGTGGTGTTCAAGGTCTCCCACAAGCCTTCTGGCC
TGGTCATGGCCAGAAAGCTAATTCATCTGGAGATCAAACCCGCAATCCGGAACCAGATCATAAGGGAGCT
GCAGGTTCTGCATGAGTGCAACTCTCCGTACATCGTGGGCTTCTATGGTGCGTTCTACAGCGATGGCGA
GATCAGTATCTGCATGGAGCACATGGATGGAGGTTCTCTGGATCAAGTCCTGAAGAAAGCTGGAAGAATT
CCTGAACAAATTTTAGGAAAAGTTAGCATTGCTGTAATAAAAGGCCTGACATATCTGAGGGAGAAGCACAA
GATCATGCACAGAGATGTCAAGCCCTCCAACATCCTAGTCAACTCCCGTGGGGAGATCAAGCTCTGTGAC
TTTGGGGTCAGCGGGCAGCTCATCGACGACATGGCCAACGACTTCGTGGGCACAAGGTCCTACATGTCG
CCAGAAAGACTCCAGGGGACTCATTACTCTGTGCAGTCAGACATCTGGAGCATGGGACTGTCTCTGGTA
GAGATGGCGGTTGGGAGGTATCCCATCCCTCCTCCAGATGCCAAGGAGCTGGAGCTGATGTTTGGGTGC
CAGGTGGAAGGAGATGCGGCTGAGACCCCACCCAGGCCAAGGACCCCCGGGAGGCCCCTTAGCTCATA
CGGAATGGACAGCCGACCTCCCATGGCAATTTTTGAGTTGTTGGATTACATAGTCAACGAGCCTCCTCCA
AAACTGCCCAGTGGAGTGTTCAGTCTGGAATTTCAAGATTTTGTGAATAAATGCTTAATAAAAAACCCCGC
AGAGAGAGCAGATTTGAAGCAACTCATGGTTCATGCTTTTATCAAGAGATCTGATGCTGAGGAAGTGGAT
TTTGCAGGTTGGCTCTGCTCCACCATCGGCCTTAACCAGCCCAGCACACCAACCCATGCTGCTGGCGTC
GGATCCACTAGTGGTTATCCGTATGATGTACCAGATTATGCAAGCCTAACTAGTTAG

Translation
MPKKKPTPAQANPAPDGSAVNGTSSAETNLEALQKKLEELELDΔΔΔΔΔΔΔΔAFLTQKQKVGELKDDDFEKISE
LGAGNGGVVFKVSHKPSGLVMARKLIHLEIKPAIRNQIIRELQVLHECNSPYIVGFYGAFYSDGEISICMEHMDG
GSLDQVLKKAGRIPEQILGKVSIAVIKGLTYLREKHKIMHRDVKPSNILVNSRGEIKLCDFGVSGQLIDDDADDFV
GTRSYMSPERLQGTHYSVQSDIWSMGLSLVEMAVGRYPIPPPDAKELELMFGCQVEGDAAETPPRPRTPGR
PLSSYGMDSRPPMAIFELLDYIVNEPPPKLPSGVFSLEFQDFVNKCLIKNPAERADLKQLMVHAFIKRSDAEEV
DFAGWLCSTIGLNQPSTPTHAAGVGSTSGYPYDVPDYASLTS-

MKK1 I9A_L11A_Δ43-51, S218D_M219D_N221D_S222D HA-tag

ERK2 – UniProt ID P28482 (part of pet28a plasmid)

DNA sequence
ATGGGCAGCAGCCATCATCATCATCATCACAGCAGCGGCCTGGTGCCGCGCGGCAGCGGTACCGAAAA
CCTGTATTTTCAGGGAGGTGGCAGCGGAGGGATGGCGGCGGCGGCGGCGGCGGGCGCAGGTCCGGA
GATGGTCCGCGGGCAGGTGTTCGACGTGGGGCCGCGCTACACTAATCTCTCGTACATCGGAGAAGGCG
CCTACGGCATGGTTTGTTCTGCTTATGATAATGTTAACAAAGTTCGAGTTGCTATCAAGAAAATCAGTCCTT
TTGAGCACCAGACCTACTGTCAGAGAACCCTGAGAGAGATAAAAATCCTACTGCGCTTCAGACATGAGAA
CATCATCGGCATCAATGACATCATCCGGGCACCAACCATTGAGCAGATGAAAGATGTATATATAGTACAG
GACCTCATGGAGACAGATCTTTACAAGCTCTTGAAGACACAGCACCTCAGCAATGATCATATCTGCTATTT
TCTTTATCAGATCCTGAGAGGATTAAAGTATATACATTCAGCTAATGTTCTGCACCGTGACCTCAAGCCTT
CCAACCTCCTGCTGAACACCACTTGTGATCTCAAGATCTGTGACTTTGGCCTTGCCCGTGTTGCAGATCC
AGACCATGATCATACAGGGTTCTTGACAGAGTATGTAGCCACGCGTTGGTACAGAGCTCCAGAAATTATG
TTGAATTCCAAGGGTTATACCAAGTCCATTGATATTTGGTCTGTGGGCTGCATCCTGGCAGAGATGCTATC
CAACAGGCCTATCTTCCCAGGAAAGCATTACCTTGACCAGCTGAATCACATCCTGGGTATTCTTGGATCT
CCATCACAGGAAGATCTGAATTGTATAATAAATTTAAAAGCTAGAAACTATTTGCTTTCTCTCCCGCACAAA
AATAAGGTGCCGTGGAACAGATTGTTCCCAAACGCTGACTCCAAAGCTCTGGATTTACTGGATAAAATGTT
GACATTTAACCCTCACAAGAGGATTGAAGTTGAACAGGCTCTGGCCCACCCGTACCTGGAGCAGTATTAT
GACCCAAGTGATGAGCCCATTGCTGAAGCACCATTCAAGTTTGACATGGAGCTGGACGACTTACCTAAGG
AGAAGCTCAAAGAACTCATTTTTGAAGAGACTGCTCGATTCCAGCCAGGATACAGATCTTAA

Translation
MGSSHHHHHHSSGLVPRGSGTENLYFQGGGSGGMAAAAAAGAGPEMVRGQVFDVGPRYTNLSYIGEGAY
GMVCSAYDNVNKVRVAIKKISPFEHQTYCQRTLREIKILLRFRHENIIGINDIIRAPTIEQMKDVYIVQDLMETDLY
KLLKTQHLSNDHICYFLYQILRGLKYIHSANVLHRDLKPSNLLLNTTCDLKICDFGLARVADPDHDHTGFLTEYV
ATRWYRAPEIMLNSKGYTKSIDIWSVGCILAEMLSNRPIFPGKHYLDQLNHILGILGSPSQEDLNCIINLKARNYL
LSLPHKNKVPWNRLFPNADSKALDLLDKMLTFNPHKRIEVEQALAHPYLEQYYDPSDEPIAEAPFKFDMELDDL
PKEKLKELIFEETARFQPGYRS-

His-tag Thrombin site TEV site ERK2

ERK_T185A_Y187A ($ERK2^{T185A/Y187A}$) (part of pet28a plasmid).[7]

DNA sequence
ATGGGCAGCAGCCATCATCATCATCATCACAGCAGCGGCCTGGTGCCGCGCGGCAGCGGTACCGAAAA
CCTGTATTTTCAGGGAGGTGGCAGCGGAGGGATGGCGGCGGCGGCGGCGGCGGGCGCAGGTCCGGA
GATGGTCCGCGGGCAGGTGTTCGACGTGGGGCCGCGCTACACTAATCTCTCGTACATCGGAGAAGGCG
CCTACGGCATGGTTTGTTCTGCTTATGATAATGTTAACAAAGTTCGAGTTGCTATCAAGAAAATCAGTCCTT
TTGAGCACCAGACCTACTGTCAGAGAACCCTGAGAGAGATAAAAATCCTACTGCGCTTCAGACATGAGAA
CATCATCGGCATCAATGACATCATCCGGGCACCAACCATTGAGCAGATGAAAGATGTATATATAGTACAG
GACCTCATGGAGACAGATCTTTACAAGCTCTTGAAGACACAGCACCTCAGCAATGATCATATCTGCTATTT
TCTTTATCAGATCCTGAGAGGATTAAAGTATATACATTCAGCTAATGTTCTGCACCGTGACCTCAAGCCTT
CCAACCTCCTGCTGAACACCACTTGTGATCTCAAGATCTGTGACTTTGGCCTTGCCCGTGTTGCAGATCC
AGACCATGATCATACAGGGTTCTTGGCGGAGGCCGTAGCCACGCGTTGGTACAGAGCTCCAGAAATTAT
GTTGAATTCCAAGGGTTATACCAAGTCCATTGATATTTGGTCTGTGGGCTGCATCCTGGCAGAGATGCTAT
CCAACAGGCCTATCTTCCCAGGAAAGCATTACCTTGACCAGCTGAATCACATCCTGGGTATTCTTGGATC
TCCATCACAGGAAGATCTGAATTGTATAATAAATTTAAAAGCTAGAAACTATTTGCTTTCTCTCCCGCACAA
AAATAAGGTGCCGTGGAACAGATTGTTCCCAAACGCTGACTCCAAAGCTCTGGATTTACTGGATAAAATG
TTGACATTTAACCCTCACAAGAGGATTGAAGTTGAACAGGCTCTGGCCCACCCGTACCTGGAGCAGTATT
ATGACCCAAGTGATGAGCCCATTGCTGAAGCACCATTCAAGTTTGACATGGAGCTGGACGACTTACCTAA
GGAGAAGCTCAAAGAACTCATTTTTGAAGAGACTGCTCGATTCCAGCCAGGATACAGATCTTAA

Translation
MGSSHHHHHHSSGLVPRGSGTENLYFQGGGSGGMAAAAAAGAGPEMVRGQVFDVGPRYTNLSYIGEGAY
GMVCSAYDNVNKVRVAIKKISPFEHQTYCQRTLREIKILLRFRHENIIGINDIIRAPTIEQMKDVYIVQDLMETDLY
KLLKTQHLSNDHICYFLYQILRGLKYIHSANVLHRDLKPSNLLLNTTCDLKICDFGLARVADPDHDHTGFLAEAV
ATRWYRAPEIMLNSKGYTKSIDIWSVGCILAEMLSNRPIFPGKHYLDQLNHILGILGSPSQEDLNCIINLKARNYL
LSLPHKNKVPWNRLFPNADSKALDLLDKMLTFNPHKRIEVEQALAHPYLEQYYDPSDEPIAEAPFKFDMELDDL
PKEKLKELIFEETARFQPGYRS-

His-tag Thrombin site TEV site ERK2 T185A_Y187A

subGFP (part of pHATa plasmid)[3,8]

DNA sequence
ATGAACACCATTCATCACCATCACCATCACAACACTAGTGGACTGAATGACATTTTCGAAGCACAGAAGAT
CGAATGGCATGAAGCCATGGGCGGTTCGGGGGGATCGGTTGCTCCATTTTCGCCGGGCGGTCGTGCAA
AAGGTGGATCAGGAGGGAGCATGGGCGGTTCGGGGGGATCGGTTGCTCCATTTTCGCCGGGCGGTCGT
GCAAAAGGTGGATCAGGAGGGAGCATGGGCGGTTCGGGGGGATCGGTTGCTCCATTTTCGCCGGGCGG
TCGTGCAAAAGGTGGATCAGGAGGGAGCATGGAAATGAGTAAAGGAGAAGAACTTTTCACTGGAGTTGT
CCCAATTCTTGTTGAATTAGATGGTGATGTTAATGGGCACAAATTTTCTGTCAGTGGAGAGGGTGAAGGT
GATGCAACATACGGAAAACTTACCCTTAAATTTATTTGCACTACTGGAAAACTACCTGTTCCTTGGCCAAC
ACTTGTCACTACTCTGACGTATGGTGTTCAATGCTTTTCCCGTTATCCGGATCACATGAAACGGCATGACT
TTTTCAAGAGTGCCATGCCCGAAGGTTATGTACAGGAACGCACTATATTCTTCAAAGATGACGGGAACTA
CAAGACGCGTGCTGAAGTCAAGTTTGAAGGTGATACCCTTGTTAATCGTATCGAGTTAAAAGGTATTGATT
TTAAAGAAGATGGAAACATTCTCGGACACAAACTCGAGTACAACTATAACTCACACAATGTATACATCACG
GCAGACAAACAAAAGAATGGAATCAAAGCTAACTTCAAAATTCGCCACAACATTGAAGATGGTTCCGTTCA
ACTAGCAGACCATTATCAACAAAATACTCCAATTGGCGATGGCCCTGTCCTTTTACCAGACAACCATTACC
TGTCGACACAATCTGCCCTTTCGAAAGATCCCAACGAAAAGCGTGACCACATGGTCCTTCTTGAGTTTGT
AACTGCTGCTGGGATTACACATGGCATGGATGAGCTCTACAAATAA

Translation
MNTIHHHHHHNTSGLNDIFEAQKIEWHEAMGGSGGSVAPFSPGGRAKGGSGGSMGGSGGSVAPFSPGGRA
KGGSGGSMGGSGGSVAPFSPGGRAKGGSGGSMEMSKGEELFTGVVPILVELDGDVNGHKFSVSGEGEGD
ATYGKLTLKFICTTGKLPVPWPTLVTTLTYGVQCFSRYPDHMKRHDFFKSAMPEGYVQERTIFFKDDGNYKTR
AEVKFEGDTLVNRIELKGIDFKEDGNILGHKLEYNYNSHNVYITADKQKNGIKANFKIRHNIEDGSVQLADHYQQ
NTPIGDGPVLLPDNHYLSTQSALSKDPNEKRDHMVLLEFVTAAGITHGMDELYK

His-tag Avi-tag Substrate tag ERK2 Superfolder GFP

FRET-Sensor (part of pOP3BT plasmid).[3,9,10]

DNA sequence
ATGAATGGACTGAATGATATCTTTGAAGCGCAGAAAATTGAATGGCATGAATCCGGATCTCATCACCATCA
CCATCACCATCACACTAGTATGGTCTCGAAAGGTGAGGAGCTCTTTACTGGCGTTGTGCCGATCTTGGTG
GAACTTGATGGCGATGTTAACGGACATAAGTTCAGCGTTAGCGGGGAAGGGGAGGGCGACGCGACCTA
CGGGAAACTGACTCTTAAATTCATCTGCACGACGGGGAAATTACCAGTCCCGTGGCCCACTTTGGTGACC
ACCTTCGGATATGGCTTAATGTGTTTTGCAAGATACCCAGATCATATGAAACAGCACGATTTCTTTAAATCT
GCGATGCCCGAAGGCTATGTGCAGGAACGAACAATCTTCTTTAAAGACGACGGAAACTATAAGACGCGC
GCGGAAGTGAAATTTGAGGGCGATACACTGGTTAATCGCATAGAGCTTAAGGGTATTGACTTCAAGGAGG
ACGGCAATATCCTCGGGCATAAACTGGAATATAACTATAATTCGCATAACGTGTATATCATGGCAGATAAA
CAGAAAAATGGAATTAAGGTTAACTTTAAAATACGCCATAATATAGAAGATGGCTCTGTCCAGCTCGCGGA
TCATTATCAGCAGAACACTCCAATTGGGGATGGACCAGTTCTTTTGCCTGATAACCATTATCTTTCTTATCA
GTCTGCGCTGTTTAAAGACCCGAACGAAAAAAGAGATCATATGGTTCTCTTAGAATTTTTGACGGCGGCA
GGTATCACCGCCATGGGCGGTTCGGGGGGATCGGTTGCTCCATTTTCGCCGGGCGGTCGTGCAAAAGG
TGGATCAGGAGGGAGCATGGAAATGAGTAAAGGAGAAGAACTTTTCACTGGAGTTGTCCCAATTCTTGTT
GAATTAGATGGTGATGTTAATGGGCACAAATTTTCTGTCAGTGGAGAGGGTGAAGGTGATGCAACATACG
GAAAACTTACCCTTAAATTTATTTGCACTACTGGAAAACTACCTGTTCCTTGGCCAACACTTGTCACTACTC
TGACGTGGGGCGTACAGTGCTTCGCGCGATATCCTGATCACATGAAACAACATGACTTTTTTAAAAGTGC
CATGCCAGAGGGCTATGTTCAGGAACGCACCATATTTTTCAAAGATGATGGCAATTATAAAACTCGCGCC
GAGGTCAAGTTTGAAGGGGATACCCTGGTAAATCGTATAGAGCTAAAAGGTATCGACTTTAAGGAGGATG
GCAATATCTTAGGCCACAAACTGGAATATAATGCGATCAGTGATAATGTGTATATCACCGCGGACAAACAA
AAAAATGGAATTAAAGCGAACTTTAAAATTCGGCACAACATCGAGGATGGATCAGTGCAGTTAGCGGATC
ATTACCAGCAGAACACTCCGATTGGTGATGGCCCAGTGTTGCTGCCTGATAACCATTATCTGTCCACGCA
GTCGGCCCTTTTTAAAGACCCGAATGAGAAACGAGATCATATGGTGTTATTGGAGTTTTTAACCGCAGCG
GGGATTACGGGCTCGAGCTAA

Translation
MNGLNDIFEAQKIEWHESGSHHHHHHHHTSMVSKGEELFTGVVPILVELDGDVNGHKFSVSGEGEGDATYGK
LTLKFICTTGKLPVPWPTLVTTFGYGLMCFARYPDHMKQHDFFKSAMPEGYVQERTIFFKDDGNYKTRAEVKF
EGDTLVNRIELKGIDFKEDGNILGHKLEYNYNSHNVYIMADKQKNGIKVNFKIRHNIEDGSVQLADHYQQNTPIG
DGPVLLPDNHYLSYQSALFKDPNEKRDHMVLLEFLTAAGITAMGGSGGSVAPFSPGGRAKGGSGGSMEMSK
GEELFTGVVPILVELDGDVNGHKFSVSGEGEGDATYGKLTLKFICTTGKLPVPWPTLVTTLTWGVQCFARYPD
HMKQHDFFKSAMPEGYVQERTIFFKDDGNYKTRAEVKFEGDTLVNRIELKGIDFKEDGNILGHKLEYNAISDNV
YITADKQKNGIKANFKIRHNIEDGSVQLADHYQQNTPIGDGPVLLPDNHYLSTQSALFKDPNEKRDHMVLLEFL
TAAGITGSS-

Avi-tag His-tag Substrate tag ERK2 Citrine Cerelean

Supplementary Table 5. Sequence of Oligonucleotides used in this study.

Name	5'-mod	Sequence
LMB_Cy5[1]	Cy5	ATGTGCTGCAAGGCGATTAAG
LMB_TxRd[1]	TxR	ATGTGCTGCAAGGCGATTAAG
T7T_DBCO[1,2]	DBCO	GCTAGTTATTGCTCAGCGG
FL_Rec_F		GATAACAATTCCCCTCTAGAAATAATTTTGTTTAACTTTAAGAAGGAGATATACA
FL_Rec_R		GTGGTGGTGACTAGTTAGGCTTGCATAATCTGGTACA
pET28_Rec_F		CTAACTAGTCACCACCACCACCACCACT
pET28a_Rec_R		TATTTCTAGAGGGGAATTGTTATCCGCT
NGS_F		TCTAGAAATAATTTTGTTTAACTTTAAGAAGGAGATATACATATGCCC
NGS_R		CAGAGCCGTCGGGGG

[1]HPLC purified. [2]From the supplier IDT.

Supplementary Table 6. Sequences of oligonucleotides used for library synthesis by SpliMLiB.

Colours match those in **Supplementary Figure 25**.

Name	Sequence
fragP13X	
P13A_F	gtattgGCTCTTCgAACGCGGCCCCCGACGGCTCTGCAGTTAACGGGACCAGC
P13D_F	gtattgGCTCTTCgAACGATGCCCCCGACGGCTCTGCAGTTAACGGGACCAGC
P13F_F	gtattgGCTCTTCgAACTTCGCCCCCGACGGCTCTGCAGTTAACGGGACCAGC
P13G_F	gtattgGCTCTTCgAACGGCGCCCCCGACGGCTCTGCAGTTAACGGGACCAGC
P13I_F	gtattgGCTCTTCgAACATCGCCCCCGACGGCTCTGCAGTTAACGGGACCAGC
P13K_F	gtattgGCTCTTCgAACAAGGCCCCCGACGGCTCTGCAGTTAACGGGACCAGC
P13L_F	gtattgGCTCTTCgAACCTGGCCCCCGACGGCTCTGCAGTTAACGGGACCAGC
P13M_F	gtattgGCTCTTCgAACATGGCCCCCGACGGCTCTGCAGTTAACGGGACCAGC
P13V_F	gtattgGCTCTTCgAACGTGGCCCCCGACGGCTCTGCAGTTAACGGGACCAGC
P13W_F	gtattgGCTCTTCgAACTGGGCCCCCGACGGCTCTGCAGTTAACGGGACCAGC
P13Y_F	gtattgGCTCTTCgAACTACGCCCCCGACGGCTCTGCAGTTAACGGGACCAGC
T7T_DBCO	See Table S5.
fragL11X	
L11A_F	GGATCCGGTGGCAAGCTGGAGGTGCTGCTCTTCgCAGGCG
L11D_F	GGATCCGGTGGCAAGCTGGAGGTGCTGCTCTTCgCAGGAT
L11F_F	GGATCCGGTGGCAAGCTGGAGGTGCTGCTCTTCgCAGTTC
L11G_F	GGATCCGGTGGCAAGCTGGAGGTGCTGCTCTTCgCAGGGC
L11I_F	GGATCCGGTGGCAAGCTGGAGGTGCTGCTCTTCgCAGATC
L11K_F	GGATCCGGTGGCAAGCTGGAGGTGCTGCTCTTCgCAGAAG
L11M_F	GGATCCGGTGGCAAGCTGGAGGTGCTGCTCTTCgCAGATG
L11P_F	GGATCCGGTGGCAAGCTGGAGGTGCTGCTCTTCgCAGCCG
L11V_F	GGATCCGGTGGCAAGCTGGAGGTGCTGCTCTTCgCAGGTG
L11W_F	GGATCCGGTGGCAAGCTGGAGGTGCTGCTCTTCgCAGTGG
L11Y_F	GGATCCGGTGGCAAGCTGGAGGTGCTGCTCTTCgCAGTAC
L11A_R	GTTCGCCTGcGAAGAGCAGCACCTCCAGCTTGCCACCGGATCC
L11D_R	GTTATCCTGcGAAGAGCAGCACCTCCAGCTTGCCACCGGATCC
L11F_R	GTTGAACTGcGAAGAGCAGCACCTCCAGCTTGCCACCGGATCC
L11G_R	GTTGCCCTGcGAAGAGCAGCACCTCCAGCTTGCCACCGGATCC
L11I_R	GTTGATCTGcGAAGAGCAGCACCTCCAGCTTGCCACCGGATCC
L11K_R	GTTCTTCTGcGAAGAGCAGCACCTCCAGCTTGCCACCGGATCC
L11M_R	GTTCATCTGcGAAGAGCAGCACCTCCAGCTTGCCACCGGATCC
L11P_R	GTTCGGCTGcGAAGAGCAGCACCTCCAGCTTGCCACCGGATCC
L11V_R	GTTCACCTGcGAAGAGCAGCACCTCCAGCTTGCCACCGGATCC
L11W_R	GTTCCACTGcGAAGAGCAGCACCTCCAGCTTGCCACCGGATCC
L11Y_R	GTTGTACTGcGAAGAGCAGCACCTCCAGCTTGCCACCGGATCC
frag(A8a)_I9X	
8aΔ_I9A_F	GGATCCGGTGGCAAGCTGGAGGTGCTGCTCTTCgCCGGCG
8aΔ_I9D_F	GGATCCGGTGGCAAGCTGGAGGTGCTGCTCTTCgCCGGAT
8aΔ_I9F_F	GGATCCGGTGGCAAGCTGGAGGTGCTGCTCTTCgCCGTTC
8aΔ_I9G_F	GGATCCGGTGGCAAGCTGGAGGTGCTGCTCTTCgCCGGGC
8aΔ_I9K_F	GGATCCGGTGGCAAGCTGGAGGTGCTGCTCTTCgCCGAAG
8aΔ_I9L_F	GGATCCGGTGGCAAGCTGGAGGTGCTGCTCTTCgCCGCTG
8aΔ_I9M_F	GGATCCGGTGGCAAGCTGGAGGTGCTGCTCTTCgCCGATG
8aΔ_I9P_F	GGATCCGGTGGCAAGCTGGAGGTGCTGCTCTTCgCCGCCG
8aΔ_I9V_F	GGATCCGGTGGCAAGCTGGAGGTGCTGCTCTTCgCCGGTG
8aΔ_I9W_F	GGATCCGGTGGCAAGCTGGAGGTGCTGCTCTTCgCCGTGG
8aΔ_I9Y_F	GGATCCGGTGGCAAGCTGGAGGTGCTGCTCTTCgCCGTAC
8aΔ_I9A_R	CTGCGCCGGcGAAGAGCAGCACCTCCAGCTTGCCACCGGATCC
8aΔ_I9D_R	CTGATCCGGcGAAGAGCAGCACCTCCAGCTTGCCACCGGATCC
8aΔ_I9F_R	CTGGAACGGcGAAGAGCAGCACCTCCAGCTTGCCACCGGATCC
8aΔ_I9G_R	CTGGCCCGGcGAAGAGCAGCACCTCCAGCTTGCCACCGGATCC
8aΔ_I9K_R	CTGCTTCGGcGAAGAGCAGCACCTCCAGCTTGCCACCGGATCC

8aΔ_I9L_R	CTGCAGCGGcGAAGAGCAGCACCTCCAGCTTGCCACCGGATCC
8aΔ_I9M_R	CTGCATCGGcGAAGAGCAGCACCTCCAGCTTGCCACCGGATCC
8aΔ_I9P_R	CTGCGGCGGcGAAGAGCAGCACCTCCAGCTTGCCACCGGATCC
8aΔ_I9V_R	CTGCACCGGcGAAGAGCAGCACCTCCAGCTTGCCACCGGATCC
8aΔ_I9W_R	CTGCCACGGcGAAGAGCAGCACCTCCAGCTTGCCACCGGATCC
8aΔ_I9Y_R	CTGGTACGGcGAAGAGCAGCACCTCCAGCTTGCCACCGGATCC
8aA_I9A_F	GGATCCGGTGGCAAGCTGGAGGTGCTGCTCTTCgCCGGCGGCG
8aA_I9D_F	GGATCCGGTGGCAAGCTGGAGGTGCTGCTCTTCgCCGGCGGAT
8aA_I9F_F	GGATCCGGTGGCAAGCTGGAGGTGCTGCTCTTCgCCGGCGTTC
8aA_I9G_F	GGATCCGGTGGCAAGCTGGAGGTGCTGCTCTTCgCCGGCGGGC
8aA_I9K_F	GGATCCGGTGGCAAGCTGGAGGTGCTGCTCTTCgCCGGCGAAG
8aA_I9L_F	GGATCCGGTGGCAAGCTGGAGGTGCTGCTCTTCgCCGGCGCTG
8aA_I9M_F	GGATCCGGTGGCAAGCTGGAGGTGCTGCTCTTCgCCGGCGATG
8aA_I9P_F	GGATCCGGTGGCAAGCTGGAGGTGCTGCTCTTCgCCGGCGCCG
8aA_I9V_F	GGATCCGGTGGCAAGCTGGAGGTGCTGCTCTTCgCCGGCGGTG
8aA_I9W_F	GGATCCGGTGGCAAGCTGGAGGTGCTGCTCTTCgCCGGCGTGG
8aA_I9Y_F	GGATCCGGTGGCAAGCTGGAGGTGCTGCTCTTCgCCGGCGTAC
8aA_I9A_R	CTGCGCCGCCGGcGAAGAGCAGCACCTCCAGCTTGCCACCGGATCC
8aA_I9D_R	CTGATCCGCCGGcGAAGAGCAGCACCTCCAGCTTGCCACCGGATCC
8aA_I9F_R	CTGGAACGCCGGcGAAGAGCAGCACCTCCAGCTTGCCACCGGATCC
8aA_I9G_R	CTGGCCCGCCGGcGAAGAGCAGCACCTCCAGCTTGCCACCGGATCC
8aA_I9K_R	CTGCTTCGCCGGcGAAGAGCAGCACCTCCAGCTTGCCACCGGATCC
8aA_I9L_R	CTGCAGCGCCGGcGAAGAGCAGCACCTCCAGCTTGCCACCGGATCC
8aA_I9M_R	CTGCATCGCCGGcGAAGAGCAGCACCTCCAGCTTGCCACCGGATCC
8aA_I9P_R	CTGCGGCGCCGGcGAAGAGCAGCACCTCCAGCTTGCCACCGGATCC
8aA_I9V_R	CTGCACCGCCGGcGAAGAGCAGCACCTCCAGCTTGCCACCGGATCC
8aA_I9W_R	CTGCCACGCCGGcGAAGAGCAGCACCTCCAGCTTGCCACCGGATCC
8aA_I9Y_R	CTGGTACGCCGGcGAAGAGCAGCACCTCCAGCTTGCCACCGGATCC
frag(7aX)	
7aA_F	GGATCCGGTGGCAAGCTGGAGGTGCTGCTCTTCgACGGCG
7aD_F	GGATCCGGTGGCAAGCTGGAGGTGCTGCTCTTCgACGGAT
7aF_F	GGATCCGGTGGCAAGCTGGAGGTGCTGCTCTTCgACGTTC
7aG_F	GGATCCGGTGGCAAGCTGGAGGTGCTGCTCTTCgACGGGC
7aI_F	GGATCCGGTGGCAAGCTGGAGGTGCTGCTCTTCgACGATC
7aK_F	GGATCCGGTGGCAAGCTGGAGGTGCTGCTCTTCgACGAAG
7aM_F	GGATCCGGTGGCAAGCTGGAGGTGCTGCTCTTCgACGATG
7aP_F	GGATCCGGTGGCAAGCTGGAGGTGCTGCTCTTCgACGCCG
7aV_F	GGATCCGGTGGCAAGCTGGAGGTGCTGCTCTTCgACGGTG
7aW_F	GGATCCGGTGGCAAGCTGGAGGTGCTGCTCTTCgACGTGG
7aY_F	GGATCCGGTGGCAAGCTGGAGGTGCTGCTCTTCgACGTAC
7aΔ_F	GGATCCGGTGGCAAGCTGGAGGTGCTGCTCTTCgACG
7aA_R	CGGCGCCGTcGAAGAGCAGCACCTCCAGCTTGCCACCGGATCC
7aD_R	CGGATCCGTcGAAGAGCAGCACCTCCAGCTTGCCACCGGATCC
7aF_R	CGGGAACGTcGAAGAGCAGCACCTCCAGCTTGCCACCGGATCC
7aG_R	CGGGCCCGTcGAAGAGCAGCACCTCCAGCTTGCCACCGGATCC
7aI_R	CGGGATCGTcGAAGAGCAGCACCTCCAGCTTGCCACCGGATCC
7aK_R	CGGCTTCGTcGAAGAGCAGCACCTCCAGCTTGCCACCGGATCC
7aM_R	CGGCATCGTcGAAGAGCAGCACCTCCAGCTTGCCACCGGATCC
7aP_R	CGGCGGCGTcGAAGAGCAGCACCTCCAGCTTGCCACCGGATCC
7aV_R	CGGCACCGTcGAAGAGCAGCACCTCCAGCTTGCCACCGGATCC
7aW_R	CGGCCACGTcGAAGAGCAGCACCTCCAGCTTGCCACCGGATCC
7aY_R	CGGGTACGTcGAAGAGCAGCACCTCCAGCTTGCCACCGGATCC
7aΔ_R	CGGCGTcGAAGAGCAGCACCTCCAGCTTGCCACCGGATCC
fragP6X	
P6A_R	gttatgGCTCTTCaCGTCGCCTTCTTCTTGGGCATatgtatatctcc
P6D_R	gttatgGCTCTTCaCGTATCCTTCTTCTTGGGCATatgtatatctcc

P6F_R	gttatgGCTCTTCaCGTGAACTTCTTCTTGGGCATatgtatatctcc
P6G_R	gttatgGCTCTTCaCGTGCCCTTCTTCTTGGGCATatgtatatctcc
P6I_R	gttatgGCTCTTCaCGTGATCTTCTTCTTGGGCATatgtatatctcc
P6K_R	gttatgGCTCTTCaCGTCTTCTTCTTCTTGGGCATatgtatatctcc
P6L2_R	gttatgGCTCTTCaCGTCAGCTTCTTCTTGGGCATatgtatatctcc
P6M_R	gttatgGCTCTTCaCGTCATCTTCTTCTTGGGCATatgtatatctcc
P6V_R	gttatgGCTCTTCaCGTCACCTTCTTCTTGGGCATatgtatatctcc
P6W_R	gttatgGCTCTTCaCGTCCACTTCTTCTTGGGCATatgtatatctcc
P6Y_R	gttatgGCTCTTCaCGTGTACTTCTTCTTGGGCATatgtatatctcc
LMB_Cy5	See SI table S5

Supplementary Table 7. Protein alignment scoring matrix used for Needleman-Wunsch alignment.

	A	R	N	D	C	Q	E	G	H	I	L	K	M	F	P	S	T	W	Y	V	B	Z	X	*
**A**	5	-1	-1	-1	-1	-1	-1	-1	-1	-1	-1	-1	-1	-1	-1	-1	-1	-1	-1	-1	-1	-1	1	-1
**R**	-1	5	-1	-1	-1	-1	-1	-1	-1	-1	-1	-1	-1	-1	-1	-1	-1	-1	-1	-1	-1	-1	1	-1
**N**	-1	-1	5	-1	-1	-1	-1	-1	-1	-1	-1	-1	-1	-1	-1	-1	-1	-1	-1	-1	-1	-1	1	-1
**D**	-1	-1	-1	5	-1	-1	-1	-1	-1	-1	-1	-1	-1	-1	-1	-1	-1	-1	-1	-1	-1	-1	1	-1
**C**	-1	-1	-1	-1	5	-1	-1	-1	-1	-1	-1	-1	-1	-1	-1	-1	-1	-1	-1	-1	-1	-1	1	-1
**Q**	-1	-1	-1	-1	-1	5	-1	-1	-1	-1	-1	-1	-1	-1	-1	-1	-1	-1	-1	-1	-1	-1	1	-1
**E**	-1	-1	-1	-1	-1	-1	5	-1	-1	-1	-1	-1	-1	-1	-1	-1	-1	-1	-1	-1	-1	-1	1	-1
**G**	-1	-1	-1	-1	-1	-1	-1	5	-1	-1	-1	-1	-1	-1	-1	-1	-1	-1	-1	-1	-1	-1	1	-1
**H**	-1	-1	-1	-1	-1	-1	-1	-1	5	-1	-1	-1	-1	-1	-1	-1	-1	-1	-1	-1	-1	-1	1	-1
**I**	-1	-1	-1	-1	-1	-1	-1	-1	-1	5	-1	-1	-1	-1	-1	-1	-1	-1	-1	-1	-1	-1	1	-1
**L**	-1	-1	-1	-1	-1	-1	-1	-1	-1	-1	5	-1	-1	-1	-1	-1	-1	-1	-1	-1	-1	-1	1	-1
**K**	-1	-1	-1	-1	-1	-1	-1	-1	-1	-1	-1	5	-1	-1	-1	-1	-1	-1	-1	-1	-1	-1	1	-1
**M**	-1	-1	-1	-1	-1	-1	-1	-1	-1	-1	-1	-1	5	-1	-1	-1	-1	-1	-1	-1	-1	-1	1	-1
**F**	-1	-1	-1	-1	-1	-1	-1	-1	-1	-1	-1	-1	-1	5	-1	-1	-1	-1	-1	-1	-1	-1	1	-1
**P**	-1	-1	-1	-1	-1	-1	-1	-1	-1	-1	-1	-1	-1	-1	5	-1	-1	-1	-1	-1	-1	-1	1	-1
**S**	-1	-1	-1	-1	-1	-1	-1	-1	-1	-1	-1	-1	-1	-1	-1	5	-1	-1	-1	-1	-1	-1	1	-1
**T**	-1	-1	-1	-1	-1	-1	-1	-1	-1	-1	-1	-1	-1	-1	-1	-1	5	-1	-1	-1	-1	-1	1	-1
**W**	-1	-1	-1	-1	-1	-1	-1	-1	-1	-1	-1	-1	-1	-1	-1	-1	-1	5	-1	-1	-1	-1	1	-1
**Y**	-1	-1	-1	-1	-1	-1	-1	-1	-1	-1	-1	-1	-1	-1	-1	-1	-1	-1	5	-1	-1	-1	1	-1
**V**	-1	-1	-1	-1	-1	-1	-1	-1	-1	-1	-1	-1	-1	-1	-1	-1	-1	-1	-1	5	-1	-1	1	-1
**B**	-1	-1	-1	-1	-1	-1	-1	-1	-1	-1	-1	-1	-1	-1	-1	-1	-1	-1	-1	-1	5	-1	1	-1
**Z**	-1	-1	-1	-1	-1	-1	-1	-1	-1	-1	-1	-1	-1	-1	-1	-1	-1	-1	-1	-1	-1	5	1	-1
**X**	1	1	1	1	1	1	1	1	1	1	1	1	1	1	1	1	1	1	1	1	1	1	-1	1
*****	-1	-1	-1	-1	-1	-1	-1	-1	-1	-1	-1	-1	-1	-1	-1	-1	-1	-1	-1	-1	-1	-1	1	1

Supplementary Table 8. Bonferroni-adjusted p-values for Chi-square goodness of fit testing for detection of epistasis.

The chi-square goodness of fit was calculated to compare the observed frequencies across all possible amino acids in the listed two positions (see **Figure 4** for a display of this enrichment), against the frequencies expected from the single position preferences (**Figure 3B**) and the appropriate degrees of freedom using the scipy.stats.chisquare function. The resulting p-values were multiplied by the number of comparisons (15) to give the Bonferroni-adjusted p-values. Where the result is given as $<10^{-308}$, the resulting p-value is smaller than the smallest possible float value in Python.

**1st position**	**2nd position**	**P-value**
6	7a	$<10^{-308}$
6	8a	5.42×10^{-16}
6	9	2.47×10^{-60}
6	11	6.62×10^{-220}
6	13	1.96×10^{-22}
7a	8a	3.43×10^{-224}
7a	9	1.98×10^{-221}
7a	11	$<10^{-308}$
7a	13	5.68×10^{-91}
8a	9	$<10^{-308}$
8a	11	1.50×10^{-55}
8a	13	3.50×10^{-45}
9	11	3.77×10^{-151}
9	13	7.70×10^{-96}
11	13	1.92×10^{-181}

Supplementary References

1. Diamante, L., Gatti-Lafranconi, P., Schaerli, Y. & Hollfelder, F. In vitro affinity screening of protein and peptide binders by megavalent bead surface display. *Protein Eng. Des. Sel.* **26**, 713–724 (2013).
2. Lindenburg, L. *et al.* Split & mix assembly of DNA libraries for ultrahigh throughput on-bead screening of functional proteins. *Nucleic Acids Res.* (2020).
3. Rodems, S. M. *et al.* A FRET-based assay platform for ultra-high density drug screening of protein kinases and phosphatases. *Assay Drug Dev. Technol.* **1**, 9–19 (2002).
4. Garai, Á. *et al.* Specificity of linear motifs that bind to a common mitogen-activated protein kinase docking groove. *Sci. Signal.* **5**, (2012).
5. Mansour, S. J., Candia, J. M., Matsuura, J. E., Manning, M. C. & Ahn, N. G. Interdependent Domains Controlling the Enzymatic Activity of Mitogen-Activated Protein Kinase Kinase 1 †. **2960**, 15529–15536 (1996).
6. Xu, B., Stippec, S., Robinson, F. L. & Cobb, M. H. Hydrophobic as well as charged residues in both MEK1 and ERK2 are important for their proper docking. *J. Biol. Chem.* **276**, 26509–26515 (2001).
7. Arya, S. B., Kumar, G., Kaur, H., Kaur, A. & Tuli, A. ARL11 regulates lipopolysaccharide-stimulated macrophage activation by promoting mitogen-activated protein kinase (MAPK) signaling. *J. Biol. Chem.* **293**, 9892–9909 (2018).
8. Pédelacq, J.-D., Cabantous, S., Tran, T., Terwilliger, T. C. & Waldo, G. S. Engineering and characterization of a superfolder green fluorescent protein. *Nat. Biotechnol.* **24**, 79–88 (2006).
9. Griesbeck, O., Baird, G. S., Campbell, R. E., Zacharias, D. A. & Tsien, R. Y. Reducing the environmental sensitivity of yellow fluorescent protein mechanism and applications. *J. Biol. Chem.* **276**, 29188–29194 (2001).
10. Rizzo, M. A. & Piston, D. W. High-contrast imaging of fluorescent protein FRET by fluorescence polarization microscopy. *Biophys. J.* **88**, L14--L16 (2005).
