# Supplementary material for: Droplet-based screening of phosphate transfer catalysis reveals how epistasis shapes MAP kinase interactions with substrates
Source: Nat Commun. 2022 Feb 11;13:844. doi: 10.1038/s41467-022-28396-4 (PMC8837617; doi:10.1038/s41467-022-28396-4)
Supplement: Supplementary file 3 — Reporting Summary [file 41467_2022_28396_MOESM3_ESM.pdf]

nature research

Corresponding author(s): F Hollfelder

Last updated by author(s): Nov 23, 2021

Reporting Summary

Nature Research wishes to improve the reproducibility of the work that we publish. This form provides structure for consistency and transparency in reporting. For further information on Nature Research policies, see our Editorial Policies and the Editorial Policy Checklist.

Statistics

For all statistical analyses, confirm that the following items are present in the figure legend, table legend, main text, or Methods section.

n/a	Confirmed	
☐	☒	The exact sample size (n) for each experimental group/condition, given as a discrete number and unit of measurement
☐	☒	A statement on whether measurements were taken from distinct samples or whether the same sample was measured repeatedly
☐	☒	The statistical test(s) used AND whether they are one- or two-sided<br>*Only common tests should be described solely by name; describe more complex techniques in the Methods section.*
☒	☐	A description of all covariates tested
☒	☐	A description of any assumptions or corrections, such as tests of normality and adjustment for multiple comparisons
☐	☒	A full description of the statistical parameters including central tendency (e.g. means) or other basic estimates (e.g. regression coefficient) AND variation (e.g. standard deviation) or associated estimates of uncertainty (e.g. confidence intervals)
☐	☒	For null hypothesis testing, the test statistic (e.g. F, t, r) with confidence intervals, effect sizes, degrees of freedom and P value noted<br>*Give P values as exact values whenever suitable.*
☒	☐	For Bayesian analysis, information on the choice of priors and Markov chain Monte Carlo settings
☒	☐	For hierarchical and complex designs, identification of the appropriate level for tests and full reporting of outcomes
☒	☐	Estimates of effect sizes (e.g. Cohen's d, Pearson's r), indicating how they were calculated

Our web collection on statistics for biologists contains articles on many of the points above.

Software and code

Policy information about availability of computer code

Data collection	FlowJo version 10.6.1 was used to collect the flow cytometry data
Data analysis	The NGS data (ENA accession PRJEB39786) were processed using dedicated code that is freely available at www.github.com/fhlab/Kinases. The reads were merged using the PEAR package v.0.9.8 and aligned with the Needleman-Wunsch algorithm implemented in the EMBOSS suite v.6.6.0. The statistics were generated with Python v.3.7.3 in the JupyterLab environment, using the packages leidenalg v.0.8.0, logomaker 0.8, network 2.4, numpy 1.18.5 and pandas 1.2.4 amongst others. The complete list of software packages used in the analysis is available in the Conda environment file available at www.github.com/fhlab/Kinases. The sequence similarity network was visualised with Gephi (https://gephi.org/, version 0.9.2).

For manuscripts utilizing custom algorithms or software that are central to the research but not yet described in published literature, software must be made available to editors and reviewers. We strongly encourage code deposition in a community repository (e.g. GitHub). See the Nature Research guidelines for submitting code & software for further information.

Data

Policy information about availability of data

All manuscripts must include a data availability statement. This statement should provide the following information, where applicable:

- Accession codes, unique identifiers, or web links for publicly available datasets
- A list of figures that have associated raw data
- A description of any restrictions on data availability

The D-domain sequences were recovered from the sorted beads with PCR-amplification as described in the Methods section. The amplified D-domains were multiplexed and processed into Illumina TruSeq libraries by the University of Cambridge, Department of Biochemistry Sequencing Facility according to the manufacturer's instructions. The sequencing data was generated by sequencing the libraries with two Illumina MiSeq 2×75 bp runs using 20% PhiX spike-in.

PDB: 4H3Q [https://www.rcsb.org/structure/4H3Q]
UNIPROT ID P28482 [https://www.uniprot.org/uniprot/P28482]
UNIPROT ID Q02750 [https://www.uniprot.org/uniprot/Q02750])

Field-specific reporting

Please select the one below that is the best fit for your research. If you are not sure, read the appropriate sections before making your selection.

☒ Life sciences ☐ Behavioural & social sciences ☐ Ecological, evolutionary & environmental sciences

For a reference copy of the document with all sections, see nature.com/documents/nr-reporting-summary-flat.pdf

Life sciences study design

All studies must disclose on these points even when the disclosure is negative.

Sample size	- The number of randomized residues in D-domain library construction was chosen to match the screening throughput (6 positions partially randomized).<br>- The droplet based screen was established for functional D-domain enrichment in 7 independent emulsions of 1E6 beads per emulsion, so that a theoretical 3-fold library overscreening was achieved. In flow cytometry experiments, ~10,000 beads per emulsion were analysed.<br>- Library screening was accompanied by another three independent control emulsions of 1E6 beads per emulsion, analyzing ~10,000 beads per control. No controls were excluded, and together with the 7 controls previously established means that the screen is robust for at least 10 unique emulsions. Likewise, we observed our nine library samples to be similar in fluorescent distribution which further exemplifies the robustness of the screening set-up.<br>- The secondary FRET assay was performed with two biological repeats for each variant, and four for each control variant. During optimization, the FRET screen was shown to be very robust across biological repeats. Unique variants were analysed in two biological repeats to justify the average activity in each gate across multiple variants.<br>- All bioinformatic analysis was done on the pool of 2.9e4 active variants.<br>- The anisotropy based direct titration of hePTP to ERK2 was done with 3 biological repeats to establish optimal concentrations of ERK2 for all subsequent competition experiments.<br>- The anisotropy based competition experiments with synthetic D-domains were performed with 2 technical repeats.
Data exclusions	See Supplementary Note 1 and Supplementary figure S11 for the full filtering steps applied in the processing of the NGS dataset. Supplementary Note 2 discusses how the filtering steps were not pre-determined, but chosen after preliminary data exploration, without affecting scientific conclusions.<br><br>For all sequences of each individual gate (Fig. 3A and 3B):<br>Choosing the cut-off values for the purpose counting variants observed in NGS:<br>- High gate: for the purpose of counting variants that are observed (but without inferring activity) we use a cut-off of 10 or more reads in the high gate, which removes the sequencing noise with some margin.<br>- Medium gate: there is a larger proportion of less well sequenced variants, so lowering the detection limit below 10 is appropriate to increase coverage. The number of detected variants stabilises at 5 reads / variant.<br>- Low gate: a similar trend to the medium gate is observed here, with the distribution of variants per read shift even more towards low sequencing counts per variant. In order to maximise the discovery of variants, we choose to use the cut-off of 3 reads per variant.<br><br>For constructing the activate dataset of MKK1 variants (Fig 3C, 4, 5 and 6):<br>We choose to build the set of active variants by focusing on the variants that show a sequencing count profile that is similar to WT or even more enriched in the high gate. Specifically, that requires:<br>- 51 or more reads in the high gate, as WT MKK1 has 51 reads; this identifies 29,603 variants.<br>- More reads in the high gate than in the medium or in the low gate (H>M and H>L count); this removes 35 variants are more abundant in the lower gates.<br>- At least 42% of reads in the high gate out of total, removing further 4 variants.<br>- No stop codons, which may occur as early PCR errors during sequencing; removes one last variant.<br>The considerations influencing active set construction are discussed in detail in Supplementary Notes 1 and 2.
Replication	Reproducibility of the droplet screen is shown in Supplementary figure S4, with additional controls in Supplementary figure S7.<br>The droplet based screen was found to enrich robustly for functional D-domains in 10 independent emulsions, with low deviation in its predictive power. Emulsion preparation was performed at least >30 times, and enrichment for functional D-domains was successful at each instance (after having established the experimental parameters of importance for set-up)
Randomization	Randomization was not relevant to this study as samples were not grouped for experiments or analyses.
Blinding	Blinding was not relevant to this study as samples were not grouped for experiments or analyses.

Reporting for specific materials, systems and methods

We require information from authors about some types of materials, experimental systems and methods used in many studies. Here, indicate whether each material, system or method listed is relevant to your study. If you are not sure if a list item applies to your research, read the appropriate section before selecting a response.

Materials & experimental systems

n/a	Involved in the study
☒	☐ Antibodies
☒	☐ Eukaryotic cell lines
☒	☐ Palaeontology and archaeology
☒	☐ Animals and other organisms
☒	☐ Human research participants
☒	☐ Clinical data
☒	☐ Dual use research of concern

Methods

n/a	Involved in the study
☒	☐ ChIP-seq
☐	☒ Flow cytometry
☒	☐ MRI-based neuroimaging

Flow Cytometry

Plots

Confirm that:

☒ The axis labels state the marker and fluorochrome used (e.g. CD4-FITC).

☒ The axis scales are clearly visible. Include numbers along axes only for bottom left plot of group (a 'group' is an analysis of identical markers).

☒ All plots are contour plots with outliers or pseudocolor plots.

☒ A numerical value for number of cells or percentage (with statistics) is provided.

Methodology

Sample preparation	Beads were washed in PBS (5% v/v tween) before analysis
Instrument	Flow cytometric analysis was carried out on a FACScan Cytek machine for beads stored in PBS (0.05% v/v Tween-20). Flow cytometric sorting of beads was performed on a BD FACSAria Fusion, with four-way sorting into different tubes according to subGFP fluorescent intensity.
Software	FlowJo version 10.6.1
Cell population abundance	All statistics for gating of the original bead population, the purity of the sample, and the final percentages that were analysed are indicated in table format beneath each individual flow cytometry plot in Fig. S1, S3, S4, S5 and S7, which contain the raw data for the flow cytometry figures shown in 2C-2H
Gating strategy	Single beads were first gated on a preliminary FSC-SSC gate. Single WT-CAMKK1 beads were gated as Cy5 positive for all control experiments. Single I9A/L11A-CAMKK1 were gated as Texas Red positive for all control experiments. Library functionalised single beads were gated as Cy5 positive, and sorted based on GFP Fluorescence. Gating strategy is shown in Figure S3, and accompanied by all statistics related to gating of the original bead population, the purity of the sample, and the final percentages that were analysed in table format beneath each individual flow cytometry plot in Supplementary Fig. S1, S3, S4, S5 and S7, which contain the raw data for the flow cytometry shown in 2C-2H

☒ Tick this box to confirm that a figure exemplifying the gating strategy is provided in the Supplementary Information.
